# Supplementary material for: Spinal microcircuits go through multiphasic homeostatic compensations in a mouse model of motoneuron degeneration
Source: Cell Rep. Author manuscript; Available in PMC 2025 Feb 23. (PMC11847574; doi:10.1016/j.celrep.2024.115046)
Supplement: 1 [file NIHMS2044448-supplement-1.pdf]

Cell Reports, Volume *43*

Supplemental information

Spinal microcircuits go through multiphasic homeostatic compensations in a mouse model of motoneuron degeneration

Filipe Nascimento, M. Görkem Özyurt, Kareen Halablab, Gardave Singh Bhumbra, Guillaume Caron, Marcin Bączyk, Daniel Zytnicki, Marin Manuel, Francesco Roselli, Rob Brownstone, and Marco Beato

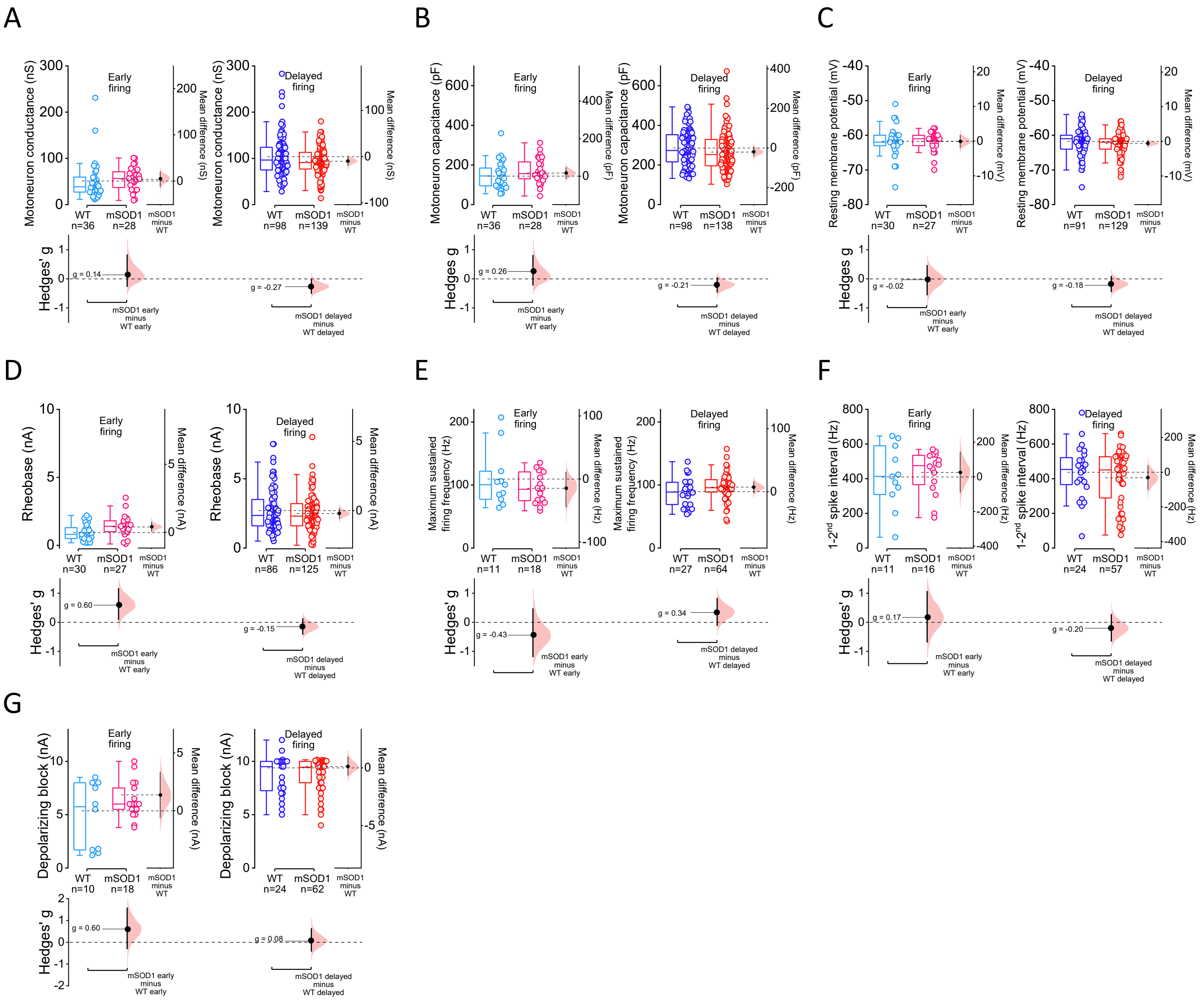

Figure S1 – Motoneuron subthreshold and repetitive firing properties from early juvenile WT and mSOD1 mice. Group data for **(A)** resting input conductance, **(B)** capacitance, **(C)** membrane potential, **(D)** rheobase, **(E)** maximum firing frequency, **(F)** 1-2nd spike interval and **(G)** depolarizing block. Estimation plots with all individual values and respective box-plots shown along with respective bootstrapped mean difference and bootstrapped Hedges' *g*. See also Table S1.

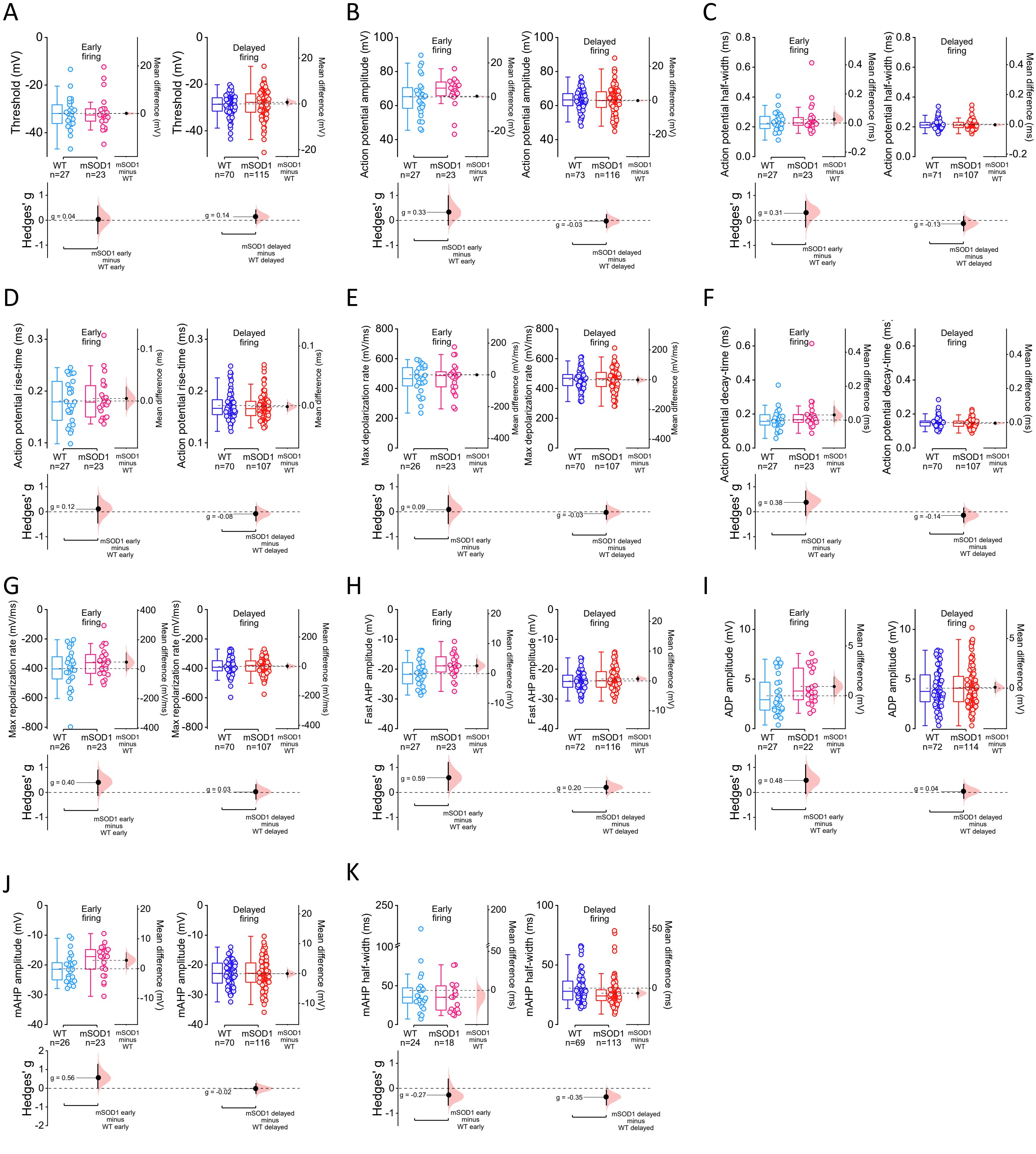

Figure S2 – Motoneuron action potential properties from early juvenile WT and mSOD1 mice. Plots for action potential **(A)** threshold, **(B)** amplitude, **(C)** half-width, **(D)** rise time, **(E)** maximum depolarization rate, **(F)** decay time, **(G)** maximum repolarization rate, **(H)** fast afterhyperpolarization (AHP) amplitude, **(I)** afterdepolarization (ADP) amplitude and medium AHP **(J)** amplitude and **(K)** half-width. Estimation plots with all individual values and respective box-plots shown along with respective bootstrapped mean difference and bootstrapped Hedges' g. See also Table S2.

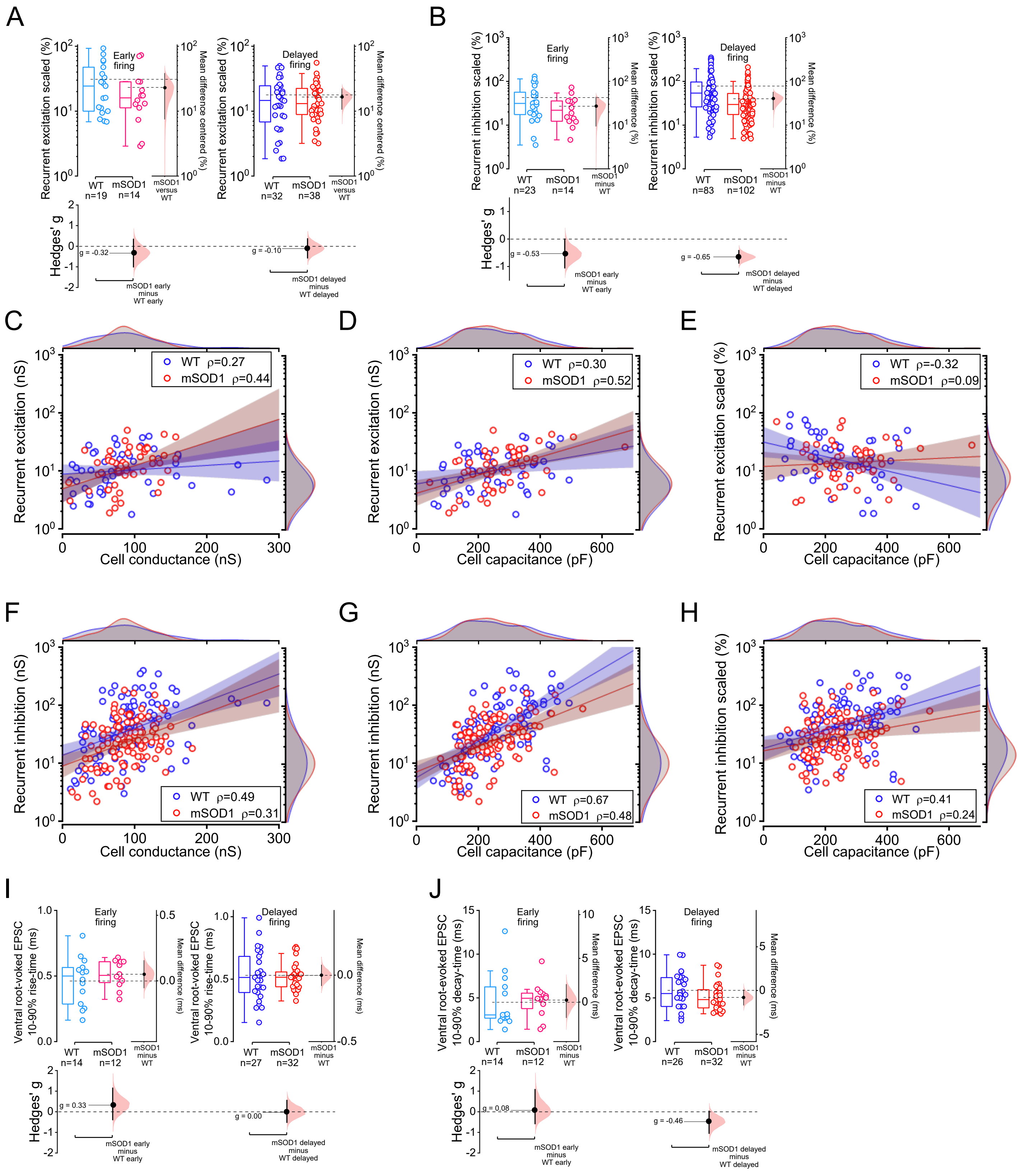

Figure S3 – Scaled recurrent synaptic conductances, correlation between synaptic conductances of recurrent circuits and cell capacitance or conductance and kinetics of ventral-root evoked currents obtained from motoneuron recordings. Group data for scaled conductances for **(A)** recurrent excitation and **(B)** recurrent inhibition. Scatter diagrams depicting the correlation between recurrent excitation and motoneuron **(C)** conductance and **(D)** capacitance, and **(E)** recurrent excitation scaled and cell capacitance. Correlation plots between synaptic conductance for recurrent inhibition and **(F)** cell conductance and **(G)** capacitance, and **(H)** recurrent inhibition scaled versus cell capacitance. Estimation plots for **(I)** rise and **(J)** decay times of ventral-root evoked excitatory EPSCs. For the correlation plots, kernel smooth (for linear scale) or lognormal (for log10 axis) distributions are shown next to respective axes, Spearman's rank-order correlation coefficient (ρ) is reported on top of the plot and linear fit (thick line) and 95% confidence interval (shaded area) are shown for visual guidance purposes only. Estimation plots with all individual values and respective box-plots shown along with respective bootstrapped mean difference and bootstrapped Hedges' g. See also Table S3.

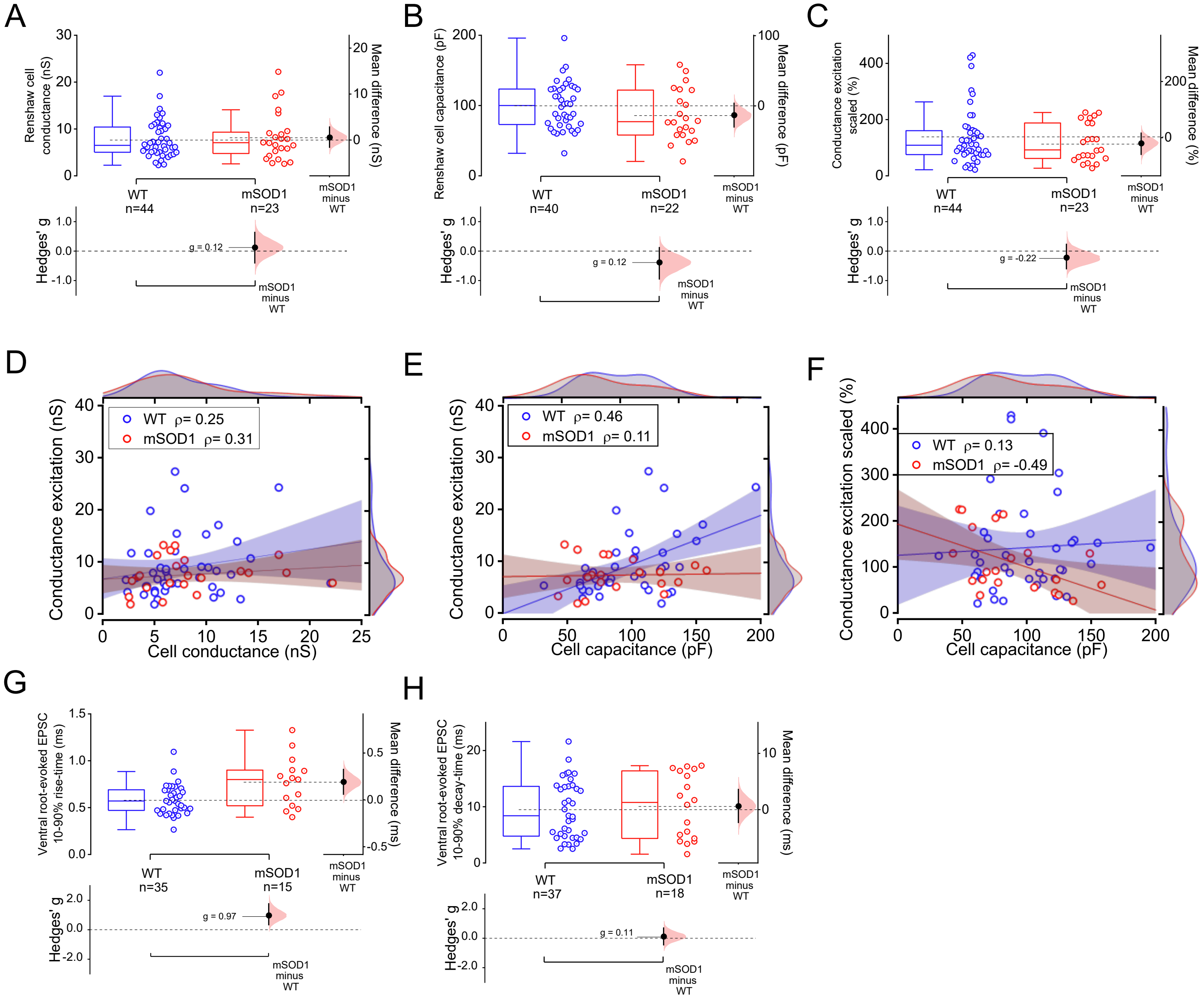

Figure S4 - Intrinsic properties of Renshaw cells, scaled synaptic conductance and correlation between cell properties and synaptic conductance and kinetics of ventral root-evoked currents. Estimation plots showing data obtained on Renshaw cell **(A)** resting conductance and **(B)** capacitance and **(C)** scaled synaptic conductance. Relationship between excitation received by Renshaw cells and **(D)** cell conductance and **(E)** capacitance and **(F)** excitation scaled against capacitance. Group data for the **(G)** rise and **(H)** decay time of the ventral root-evoked EPSCs measured from Renshaw cells from WT and mSOD1 mice. For the correlation plots, Kernel smooth distributions are shown next to respective axes, Spearman's rank-order correlation coefficient (ρ) is reported on top of the plot and linear fit (thick line) and 95% confidence interval (shaded area) are shown for visual guidance purposes only. Estimation plots with all individual values and respective box-plots shown along with respective bootstrapped mean difference and bootstrapped Hedges' g. See also Table S4.

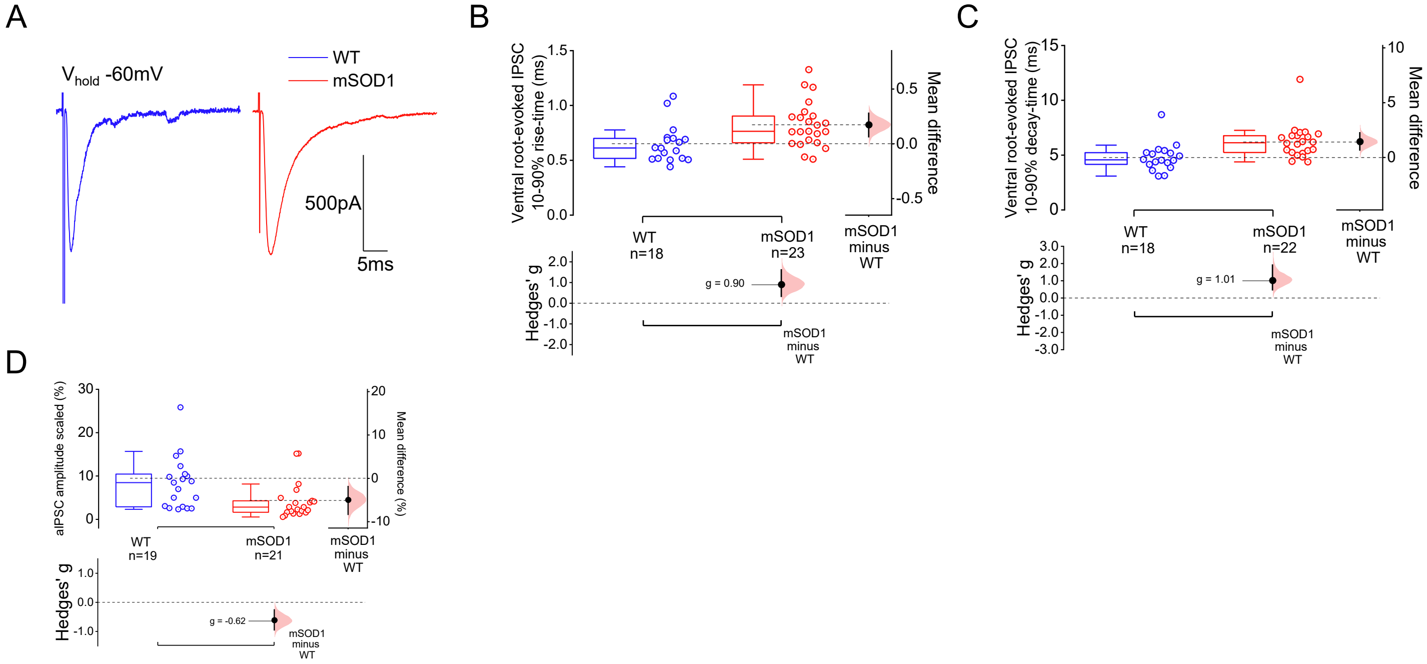

Figure S5 –Kinetics of ventral-root evoked glycinergic currents and scaled conductance from asynchronous IPSCs (aIPSCs) . (A) Example of individual IPSCs obtained from WT and mSOD1 mouse motoneurons before replacing Ca^{2+} with Sr^{2+} in the perfusion solution. Group data for IPSC **(B)** rise and **(C)** decay times. **(D)** scaled conductances from aIPSCs. Estimation plots with all individual values and respective box-plots shown along with respective bootstrapped mean difference and bootstrapped Hedges' g. Hierarchical resampling used for figure (D) with each dot representing the mean per animal. See also Table S5.

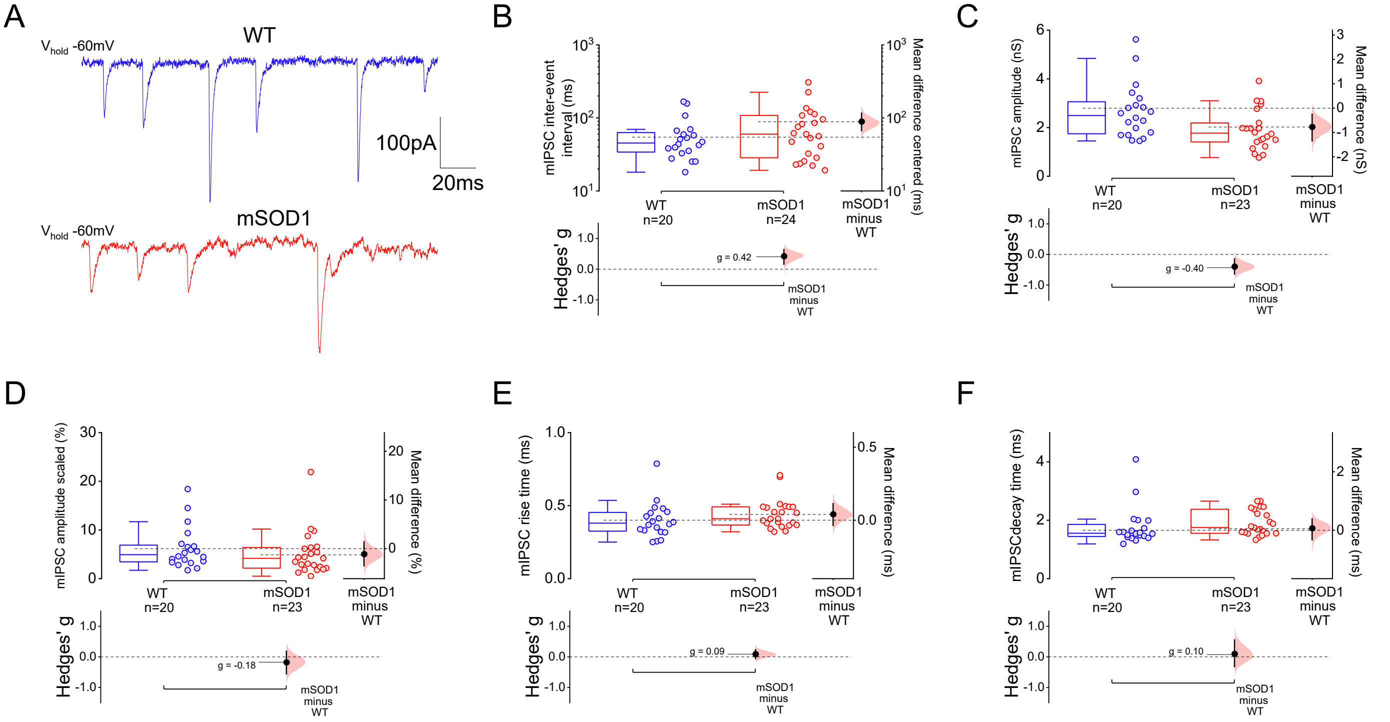

Figure S6 – Glycinergic mIPSCs are mildly affected in motoneurons from early juvenile mSOD1 mice. (A) examples of mIPSCs recorded from motoneurons from WT and mSOD1 mice. Estimation plots with data for mIPSC **(B)** inter-event interval, amplitude **(C)** absolute conductance and **(D)** scaled conductance, **(E)** rise and **(F)** decay times. Each dot represents the mean per animal, and mean difference and Hedges' *g* effect sizes were computed using hierarchical resampling. See also Table S5.

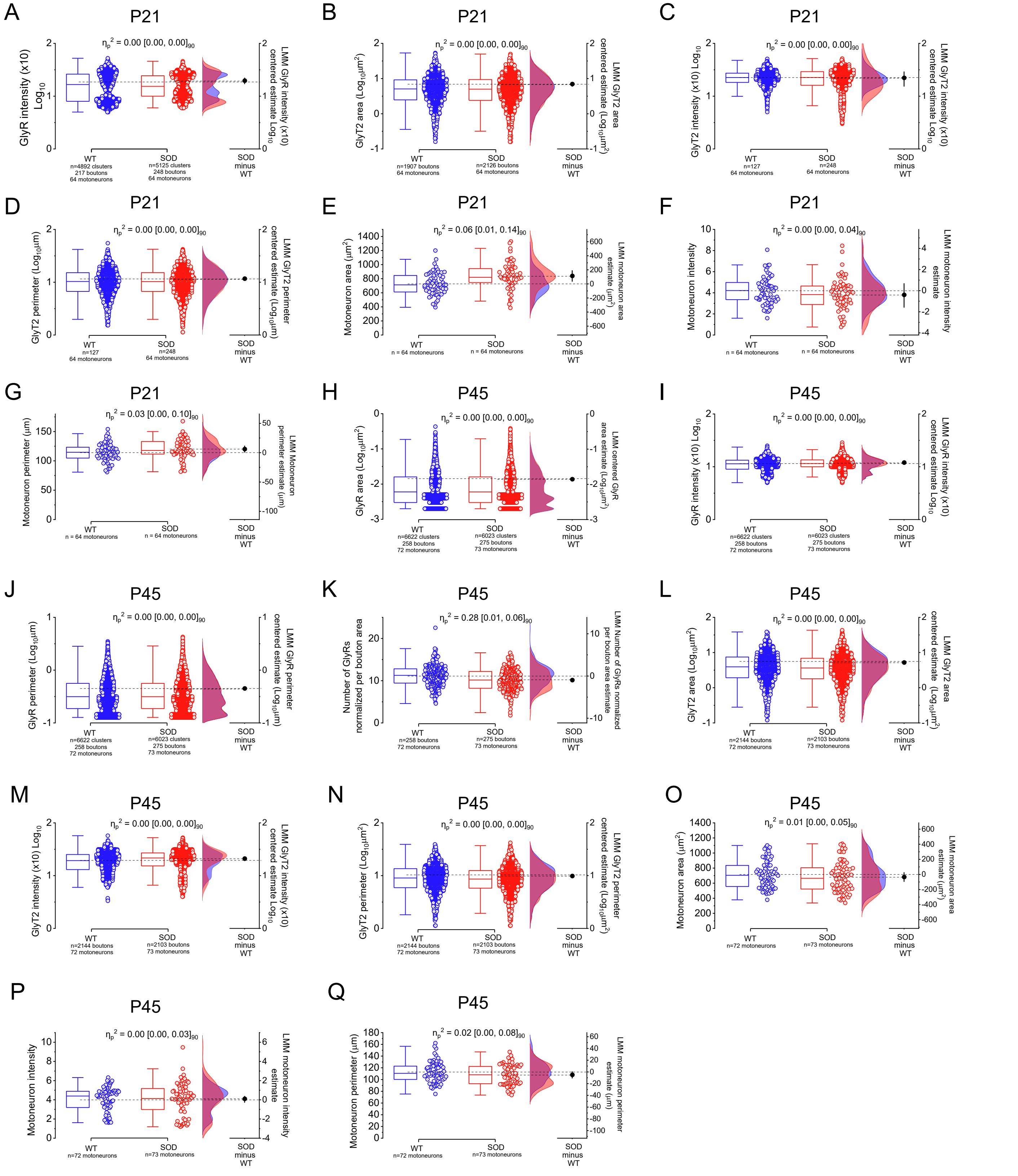

Figure S7 – Data from P21 and P45 mice immunohistochemistry. Group data from P21 mice for synaptic markers such as **(A)** GlyR intensity, GlyT2 **(B)** area, **(C)** intensity and **(D)** perimeter and motoneuron **(E)** area, **(F)** intensity and **(G)** perimeter. Group data obtained from P45 mice for GlyR **(H)** area, **(I)** number per bouton, **(J)** perimeter and **(K)** density, presynaptic GlyT2 **(L)** area, **(M)** number per bouton and **(N)** perimeter and motoneuron **(O)** area, **(P)** intensity and **(Q)** perimeter. Box-plots with all individual values and Kernel smooth distribution shown along with respective Linear Mixed Model estimates. See also Tables S6-9.

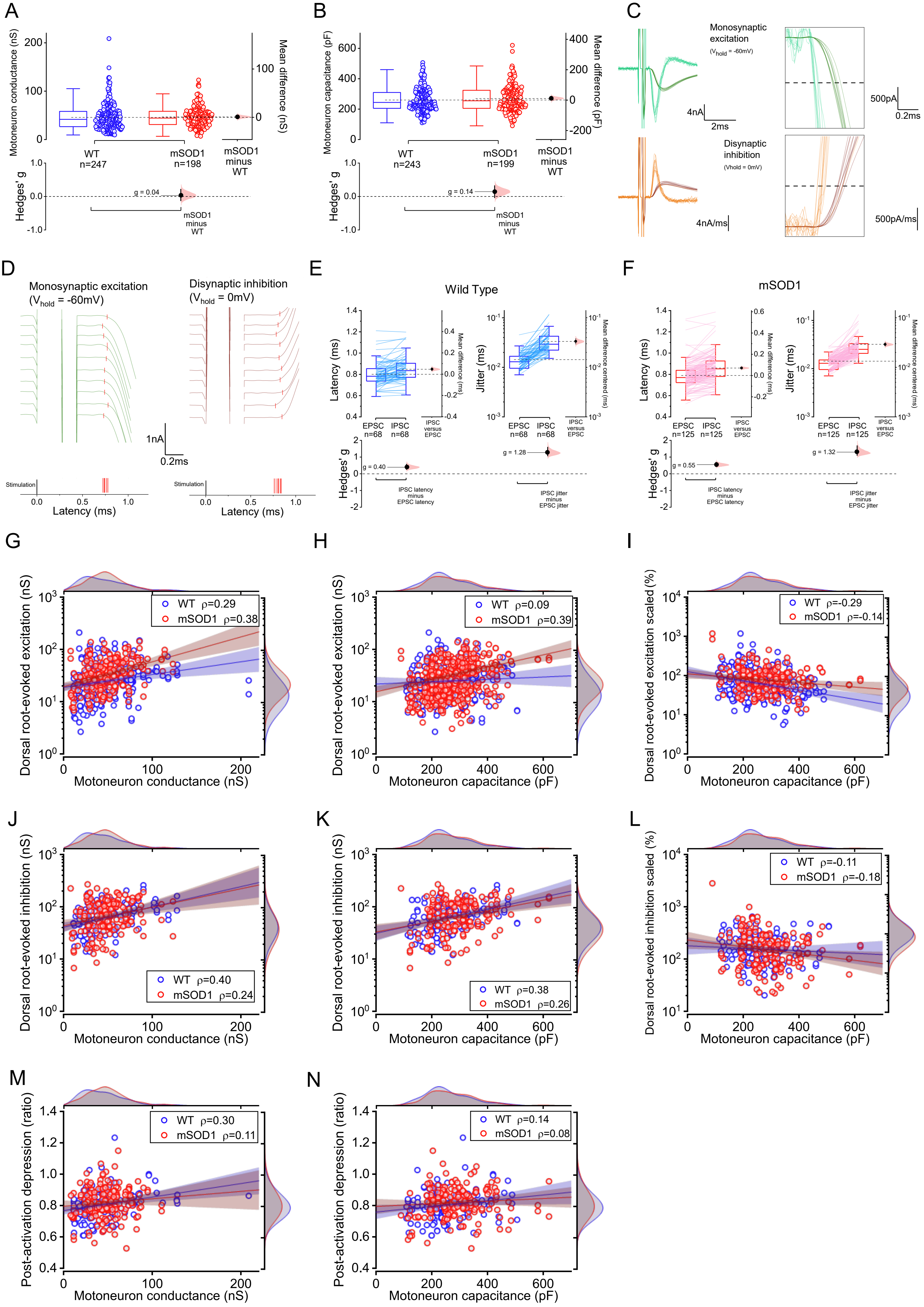

A
B
C
D
E
F
G
H
I
J
K
L
M
N
Motoneuron conductance (nS)
Mean difference (nS)
WT n=247
mSOD1 n=198
mSOD1 minus WT
Hedges' g
g = 0.04
Motoneuron capacitance (pF)
Mean difference (pF)
WT n=243
mSOD1 n=199
g = 0.14
Monosynaptic excitation (V_{hold} = -60mV)
Disynaptic inhibition (Vhold = 0mV)
4nA
2ms
4nA/ms
500pA
0.2ms
500pA/ms
Monosynaptic excitation (V_{hold} = -60mV)
Disynaptic inhibition (V_{hold} = 0mV)
1nA
0.2ms
Stimulation
Latency (ms)
Wild Type
mSOD1
Latency (ms)
Jitter (ms)
Mean difference (ms)
Mean difference centered (ms)
EPSC n=68
IPSC n=68
EPSC n=125
IPSC n=125
IPSC versus EPSC
g = 0.40
g = 1.28
g = 0.55
g = 1.32
IPSC latency minus EPSC latency
IPSC jitter minus EPSC jitter
Dorsal root-evoked excitation (nS)
Dorsal root-evoked excitation scaled (%)
Dorsal root-evoked inhibition (nS)
Dorsal root-evoked inhibition scaled (%)
Post-activation depression (ratio)
Motoneuron conductance (nS)
Motoneuron capacitance (pF)
WT ρ=0.29
mSOD1 ρ=0.38
WT ρ=0.09
mSOD1 ρ=0.39
WT ρ=-0.29
mSOD1 ρ=-0.14
WT ρ=0.40
mSOD1 ρ=0.24
WT ρ=0.38
mSOD1 ρ=0.26
WT ρ=-0.11
mSOD1 ρ=-0.18
WT ρ=0.30
mSOD1 ρ=0.11
WT ρ=0.14
mSOD1 ρ=0.08

Figure S8 – Intrinsic properties of motoneurons obtained from ventral-horn ablated in vitro spinal cords, latency and jitter of dorsal root responses and correlations between motoneuron properties and sensory-evoked synaptic conductances and post-activation depression. Motoneuron **(A)** conductance and **(B)** capacitance obtained from recordings performed from ventral horn-ablated *in vitro* spinal cords. **(C)** Examples of dorsal-root evoked EPSCs (dark green) and IPSCs (brown) with respective derivative for each trace (lighted colours), with boxes representing zoomed in sections illustrating the 5x standard deviation of the baseline noise from the derivative (dashed line) used as proxy for the onset of the synaptic current. **(D)** EPSCs and IPSCs from (C) vertically aligned, with synaptic current onset demarked by red line and latency rug at the bottom of each series of traces. Jitter and latency obtained from Wild Type **(E)** and mSOD1 **(F)** dorsal root evoked responses. Correlation plots for dorsal root-evoked excitation and **(D)** cell conductance or **(E)** capacitance, **(F)** excitation scaled and capacitance, disynaptic inhibition and **(G)** motoneuron conductance or **(H)** capacitance, inhibition scaled against capacitance **(I)** and post-activation depression ratio versus **(J)** cell conductance or **(K)** capacitance. For the correlation plots, kernel smooth (for linear scale) or lognormal (for log10 axis) distributions are shown next to respective axes, Spearman's rank-order correlation coefficient (ρ) is reported on top of the plot and linear fit (thick line) and 95% confidence interval (shaded area) are shown for visual guidance purposes only. Estimation plots with all individual values and respective box-plots shown along with respective bootstrapped mean difference and bootstrapped Hedges' g. See also Tables S11-S18.

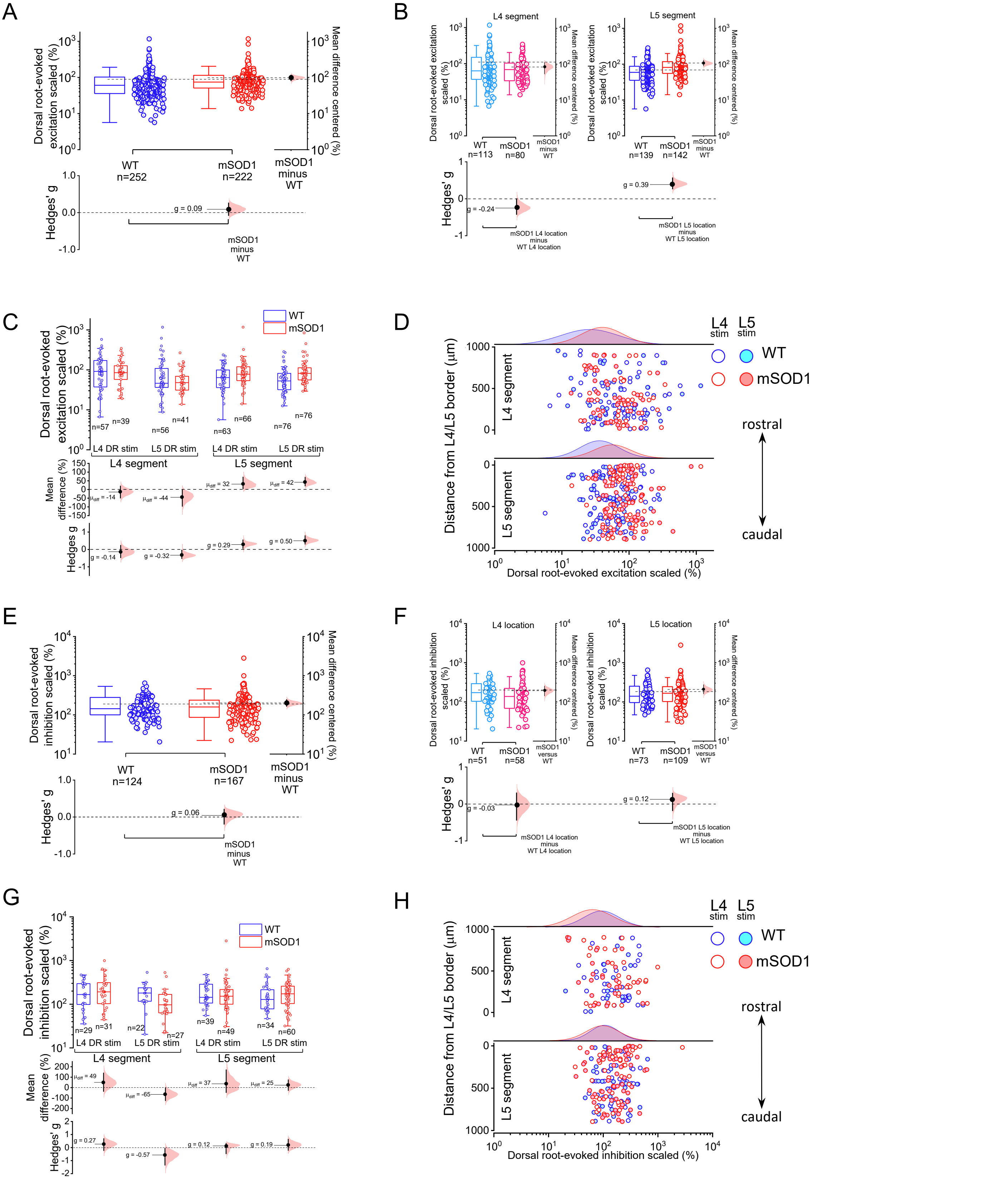

Figure S9 – Dorsal root-evoked monosynaptic (Ia) excitation and disynaptic (Ia/Ib) inhibition scaled conductances obtained from motoneuron recordings. (A) Data for monosynaptic Ia excitation scaled to resting cell conductance obtained from motoneuron recordings, **(B)** organized according to segment position and **(C)** according to stimulated root and location. **(D)** the values split by cell location (left) and with scaled conductances plotted against distance from L4/L5 border (right). Data for absolute and scaled conductance of disynaptic Ia/Ib inhibition from motoneurorganized according to segment and **(E and F)** grouped based on stimulated root and location **(G and H)**, and values organized by cell location (left) and with scaled inhibitory conductances plotted against distance from L4/L5 border (right). Estimation plots with all individual values and respective box-plots shown along with respective bootstrapped mean difference and bootstrapped Hedges' g. See also Tables S11-S17.

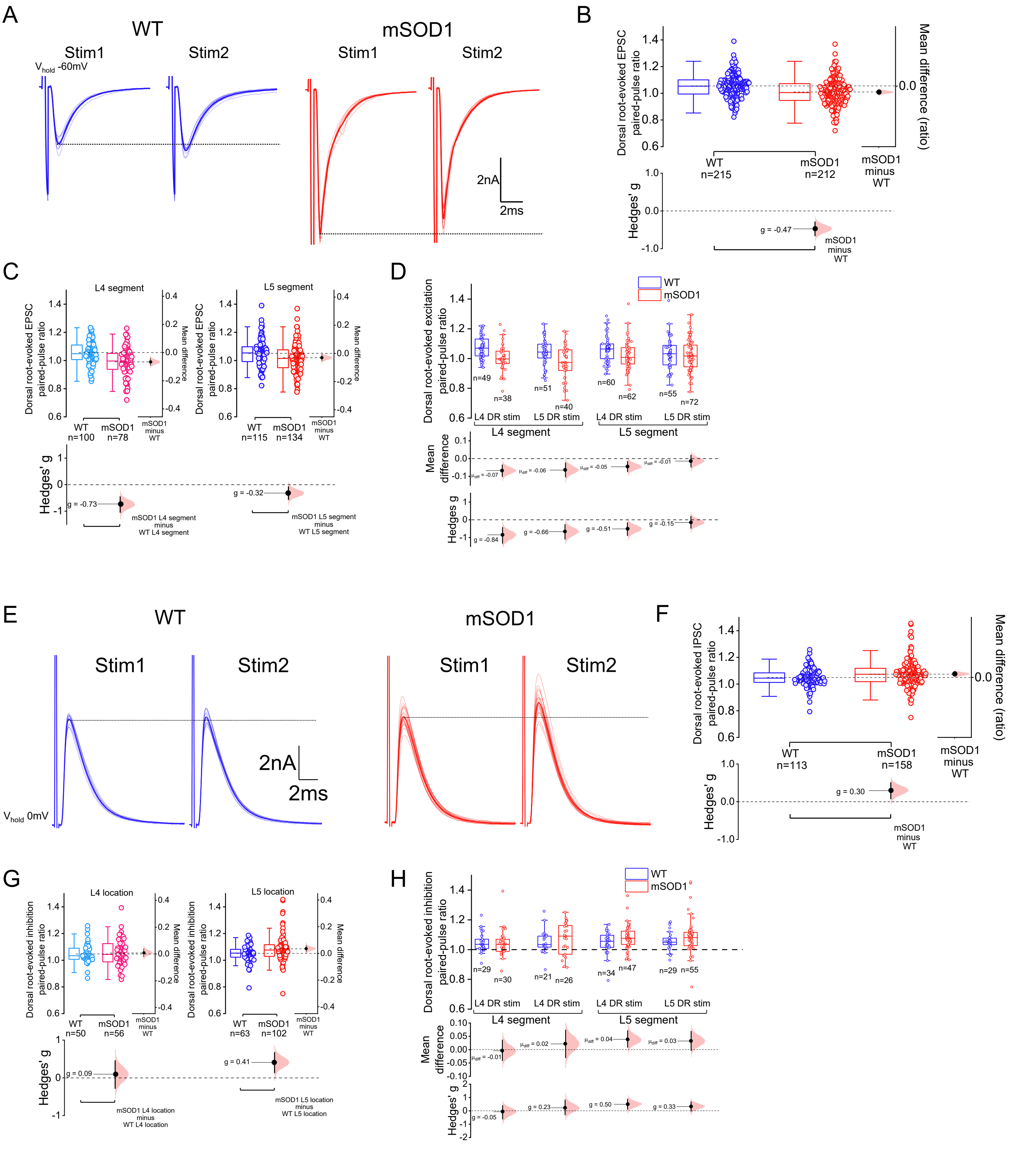

Figure S10 – Dorsal root-evoked monosynaptic (Ia) excitation and disynaptic (Ia/Ib) inhibition paired-pulse ratios from motoneuron recordings. (A) Examples of dorsal-root evoked paired pulse responses (33 Hz) for WT and mSOD1 mice, with group data for **(B)** all observations, **(C)** recordings organized per location and **(D)** ratios grouped per segment and stimulating root. **(E)** Example traces of dorsal-root evoked disynaptic inhibitory paired pulse responses (33 Hz) for control and mutant mice, with group data for **(F)** all observations, **(G)** recordings organized per anatomical position and **(H)** ratios grouped per location and stimulating root. Estimation plots with all individual values and respective box-plots shown along with respective bootstrapped mean difference and bootstrapped Hedges' g. See also Tables S11-S17.

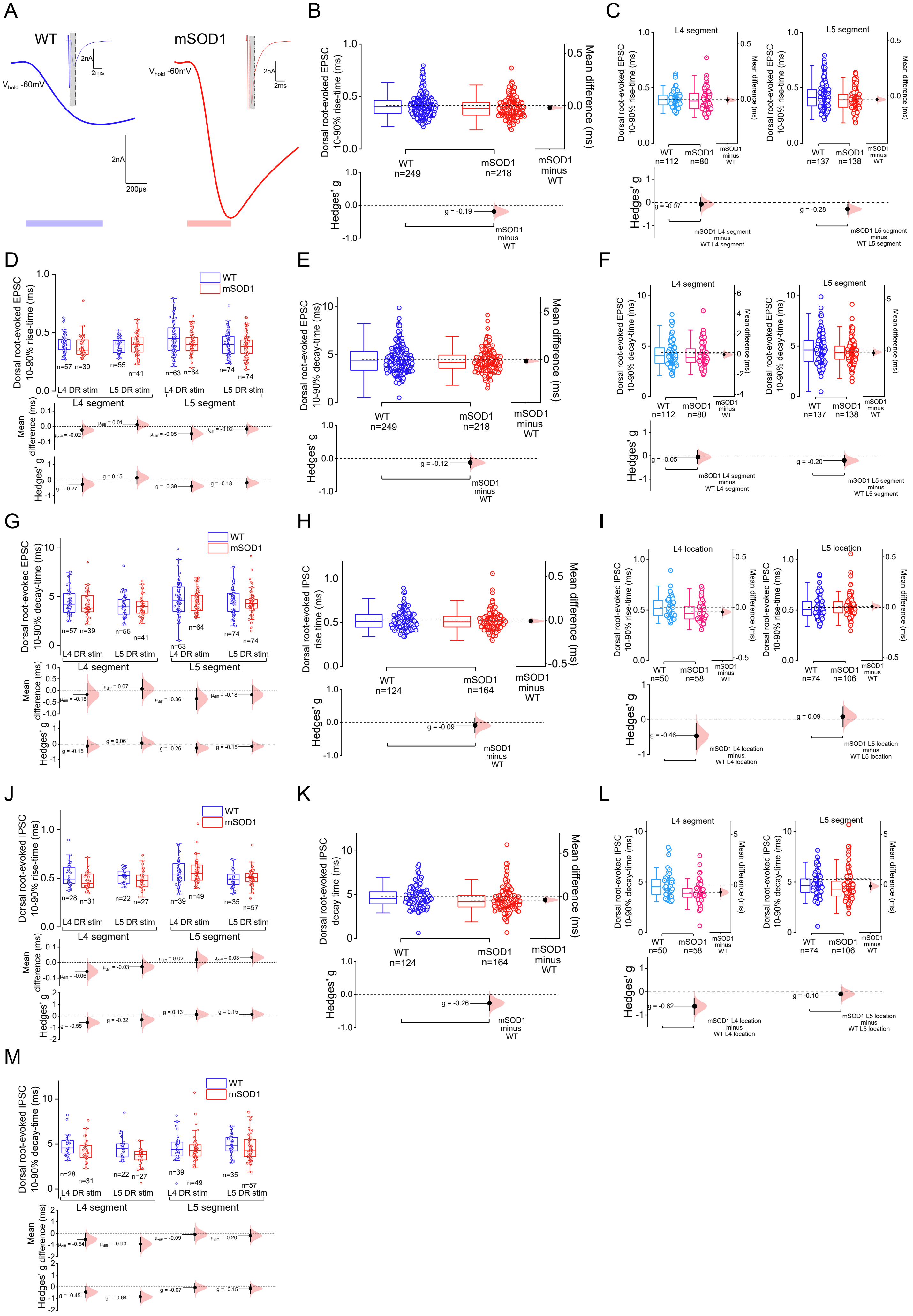

A
WT
mSOD1
V_{hold} -60mV
2nA
2ms
200μs
B
Dorsal root-evoked EPSC 10-90% rise-time (ms)
Mean difference (ms)
WT n=249
mSOD1 n=218
mSOD1 minus WT
Hedges' g
g = -0.19
C
L4 segment
L5 segment
WT n=112
mSOD1 n=80
WT n=137
mSOD1 n=138
g = -0.07
g = -0.28
mSOD1 L4 segment minus WT L4 segment
mSOD1 L5 segment minus WT L5 segment
D
L4 DR stim
L5 DR stim
E
Dorsal root-evoked EPSC 10-90% decay-time (ms)
g = -0.12
F
g = -0.05
g = -0.20
G
H
Dorsal root-evoked IPSC rise time (ms)
WT n=124
mSOD1 n=164
g = -0.09
I
L4 location
L5 location
Dorsal root-evoked IPSC 10-90% rise-time (ms)
WT n=50
mSOD1 n=58
WT n=74
mSOD1 n=106
g = -0.46
g = 0.09
mSOD1 L4 location minus WT L4 location
mSOD1 L5 location minus WT L5 location
J
K
Dorsal root-evoked IPSC decay time (ms)
g = -0.26
L
Dorsal root-evoked IPSC 10-90% decay-time (ms)
g = -0.62
g = -0.10
M

Figure S11– Dorsal root-evoked monosynaptic (Ia) EPSC and disynaptic (Ia/Ib) IPSC rise and decay times from motoneurons. (A) Close up of the initial phase of the example dorsal root-evoked EPSCs from WT and mSOD1 with the duration of the rise phase represented at the bottom. Estimation plots showing data for all root responses **(B and E)**, recordings grouped by segment **(C and F)** and results shown by location and stimulating root **(D and G)** for rise and decay phases of the dorsal-root evoked Ia monosynaptic EPSC. Group data for all disynaptic root responses **(H and K)**, recordings grouped by segment **(I and L)** and results shown by location and stimulating root **(J and M)** for rise and decay phases of the dorsal-root evoked Ia/Ib disynaptic EPSC. Estimation plots with all individual values and respective box-plots shown along with respective bootstrapped mean difference and bootstrapped Hedges' g. See also Tables S11-S17.

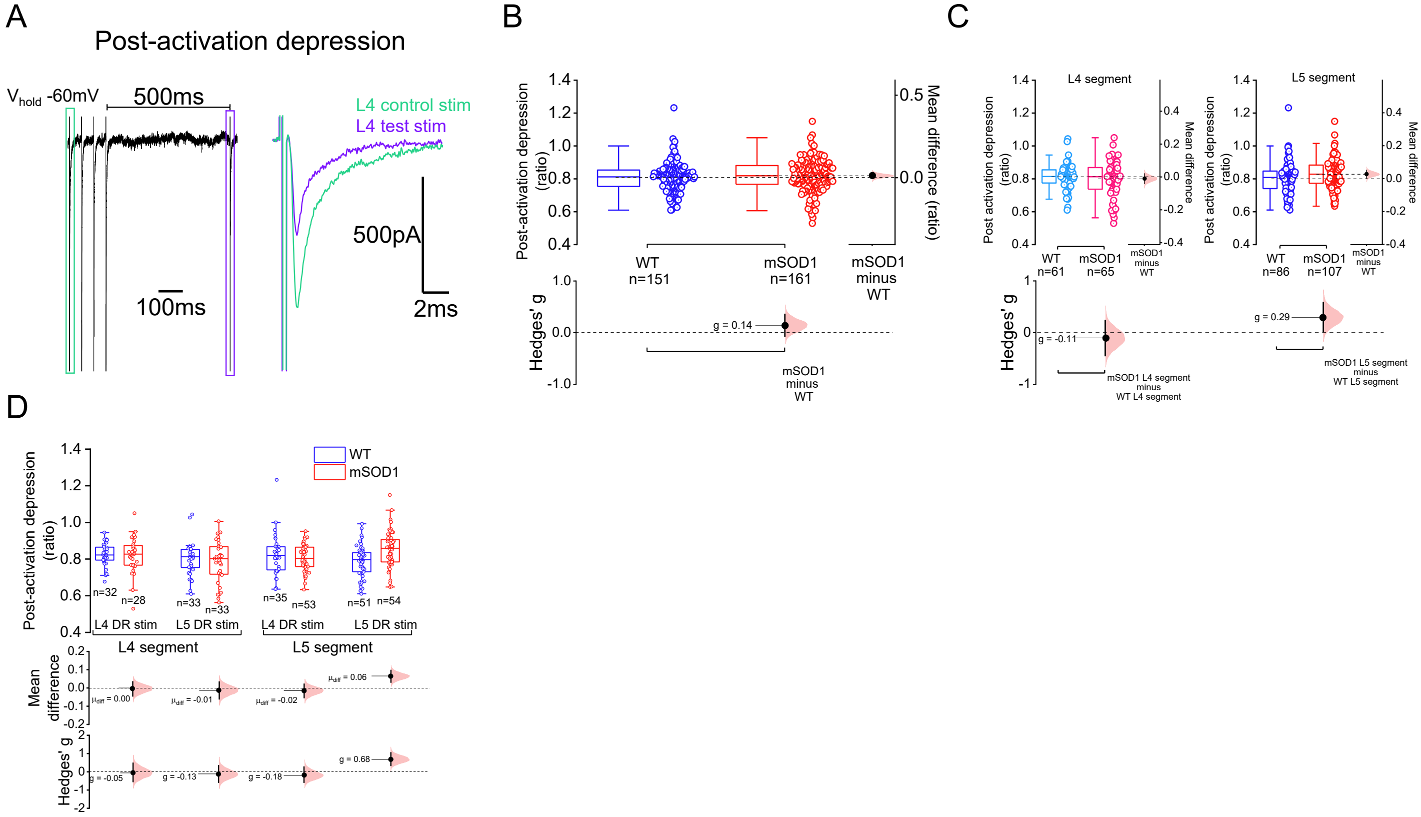

Figure S12 – Post-activation depression motoneuron responses organized according to segment location and stimulating root. (I) Example of the conditioning protocol used to test post-activation depression *in vitro*, with the amplitude of a first EPSC from a train of 4 responses at 20 Hz (L4 control stim) is compared to that of a fifth EPSC triggered 500 ms after the 4th repetitive stimuli (L4 test stim). **(J)** Group data for post-activation depression.**(A)** Data for post-activation depression grouped according to segment position and **(B)** to stimulated root and location**.** Estimation plots with all individual values and respective box-plots shown along with respective bootstrapped mean difference and bootstrapped Hedges' g. See also Tables S11-S17.

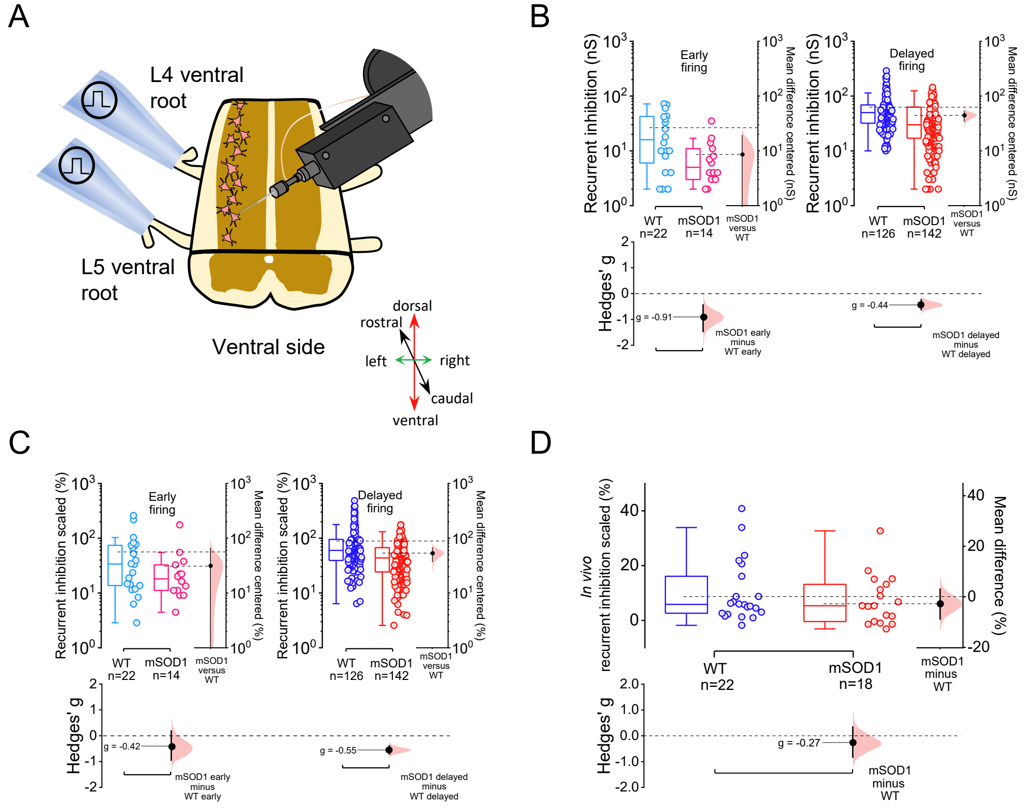

Figure S13 – *In vitro* recurrent inhibition from distal muscle-innervating lumbar motoneurons and scaled recurrent inhibition conductances from *in vivo* recordings. (A) Representation of the dorsal horn-ablated *in vitro* spinal cord preparation used to estimate recurrent inhibition from favorably targeted dorsolateral motoneurons (each *n* corresponds to a root response), with respective data obtained on **(B)** absolute and **(C)** scaled recurrent inhibition. **(D)** Group data for scaled recurrent inhibition estimated from *in vivo* motoneuron recordings. Estimation plots with all individual values and respective box-plots shown along with respective bootstrapped mean difference and bootstrapped Hedges' *g***.** See also Table S22.

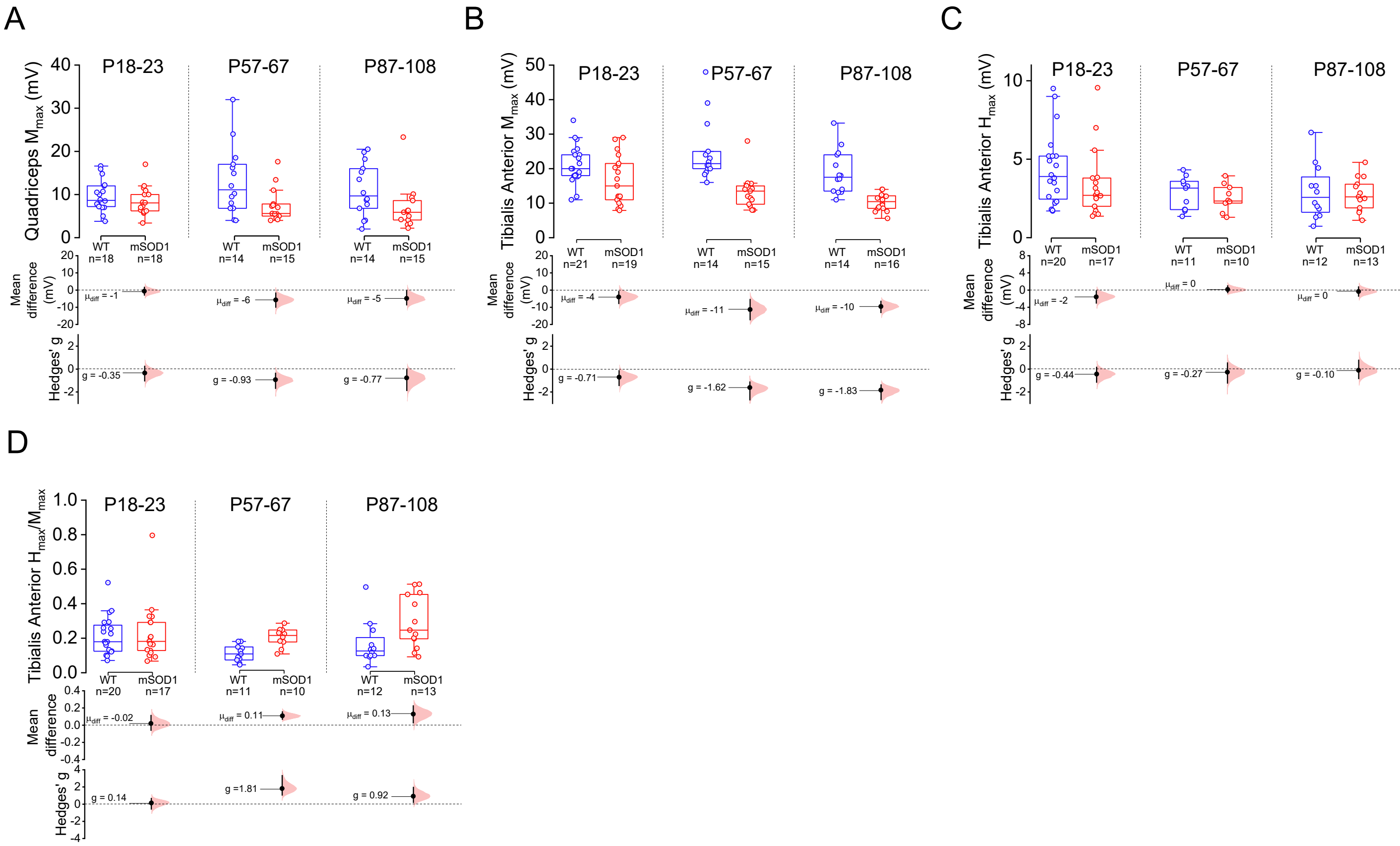

Figure S14 – M_{max} and H_{max} values obtained from *in vivo* EMG experiments. Data obtained on **(A)** quadriceps M_{max} and Tibialis Anterior **(B)** M_{max} , **(C)** H_{max} and **(D)** H_{max}/M_{max} ratio obtained from different age ranges (each *n* is an animal). Estimation plots with all individual values and respective box-plots shown along with respective bootstrapped mean difference and bootstrapped Hedges' *g*. See also Table S20.

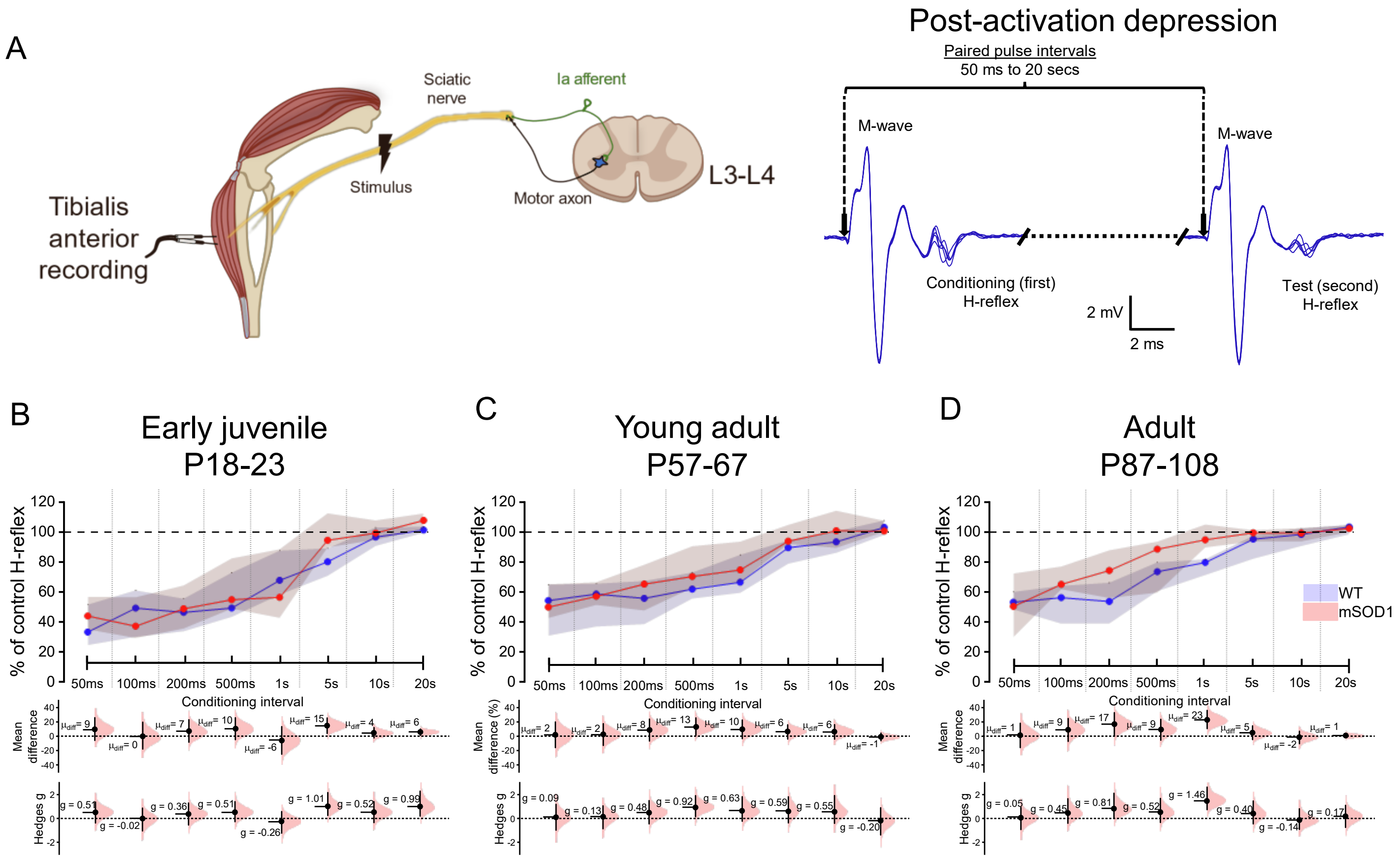

Figure S15 – Post-activation depression from *in vivo* EMG experiments. Schematic and example traces illustrating the EMG recordings used to obtain motor and H-reflex responses from tibialis anterior muscles, and the conditioning protocols used to estimate **(A)** post-activation depression. Data obtained for **(B-D)** post-activation depression (each *n* is an animal). Estimation plots with box-plots shown as median (dot) and interquartile range (shaded area) for post-activation depression along with respective bootstrapped mean difference and bootstrapped Hedges' ***g*****.** See also Table S21.

Table S1 – Mean and standard deviation, intraclass correlation coefficient (ICC), bootstrapped mean difference and Hedges' *g* for intrinsic properties and firing output from early and delayed firing motoneurons obtained from oblique slices

Early firing motoneurons						
Parameter	Mean±standard deviation		ICC		Bootstrapped Mean difference [95% CI]	Bootstrapped Hedges' *g* [95% CI]
	WT	mSOD1	WT	mSOD1		
Conductance (ns)	51±43 n=36 19 mice	56±24 n=28 15 mice	0.06	**0.65**	5 [-12, 20]	0.14 [-0.27, 0.84]
Capacitance (pF)	151±66 n=36 19 mice	168±63 n=28 15 mice	-0.32	-0.33	17 [-14, 49]	0.26 [-0.23, 0.82]
Membrane potential (mv)	-62±4 n=30 18 mice	-62±3 n=27 15 mice	**0.60**	-0.27	0 [-2, 2]	-0.02 [-0.56, 0.47]
Rheobase (nA)	0.95±0.60 n=30 17 mice	1.36±0.76 n=27 15 mice	-0.14	0.19	**0.41 [0.06, 0.77]**	**0.60 [0.08, 1.17]**
Maximum sustained firing (Hz)	110±46 n=11 5 mice	95±24 n=18 10 mice	0.00	-0.18	-15 [-45, 11]	-0.43 [-1.20, 0.48]
1-2nd spike interval (Hz)	411±181 n=11 5 mice	436±122 n=16 9 mice	**0.67**	0.16	26 [-92, 144]	0.17 [-0.69, 1.08]
Depolarizing block (nA)	4.96±3.10 n=10 5 mice	6.32±1.69 n=18 10 mice	-0.08	-0.17	1.36 [-0.63, 3.36]	0.60 [-0.31, 1.60]
Delayed firing motoneurons						
Parameter	Mean±standard deviation		ICC		Bootstrapped Mean difference [95% CI]	Bootstrapped Hedges' *g* [95% CI]
	WT	mSOD1	WT	mSOD1		
Conductance (ns)	104±44 n=98 31 mice	94±30 n=139 29 mice	0.33	0.11	-10 [-20, 0.1]	-0.27 [-0.52, 0.01]
Capacitance (pF)	286±93 n=98 31 mice	266±94 n=138 29 mice	0.05	0.40	-20 [-42, 5]	-0.21 [-0.47, 0.05]
Membrane potential (mv)	-62±4 n=91 29 mice	-63±3 n=129 28 mice	0.00	-0.04	-0.5 [-1.4, 0.3]	-0.18 [-0.46, 0.10]
Rheobase (nA)	2.69±1.54 n=86 29 mice	2.49±1.21 n=125 28 mice	0.29	0.07	-0.20 [-0.59, 0.19]	-0.15 [-0.43, 0.14]
Maximum sustained firing (Hz)	89±22 n=27 9 mice	97±20 n=64 12 mice	0.06	0.21	7 [-3, 17]	0.34 [-0.12, 0.83]
1-2nd spike interval (Hz)	437±151 n=24 9 mice	406±156 n=57 11 mice	0.21	0.19	-31 [-102, 40]	-0.20 [-0.66, 0.28]
Depolarizing block (nA)	8.67±1.86 n=24 9 mice	8.79±1.44 n=62 12 mice	0.03	0.07	0.12 [-0.66, 0.96]	0.08 [-0.43, 0.65]

CI - confidence interval; for mixed model results when ICC>0.50 see table S24

Table S2 - Mean and standard deviation, intraclass correlation coefficient (ICC), bootstrapped mean difference and Hedges' *g* for action potential properties extracted from early and delayed firing motoneuron recordings from oblique slices

Early firing motoneurons						
Parameter	Mean±standard deviation		ICC		Bootstrapped Mean difference [95% CI]	Bootstrapped Hedges' *g* [95% CI]
	WT	mSOD1	WT	mSOD1		
Threshold (mV)	-32±7 n=27 15 mice	-32±7 n=23 14 mice	0.12	0.21	0 [-1, 1]	0.04 [-0.56, 0.59]
Action potential amplitude (mV)	65±11 n=27 15 mice	68±8 n=23 14 mice	0.07	0.18	3 [-2, 9]	0.33 [-0.20, 1.01]
Action potential half-width (ms)	0.23±0.06 n=27 15 mice	0.26±0.10 n=23 14 mice	**0.58**	**0.92**	0.03 [-0.02, 0.07]	0.31 [-0.28, 0.78]
Action potential rise time (ms)	0.18±0.04 n=27 15 mice	0.19±0.04 n=23 14 mice	0.37	**0.51**	0.01 [-0.02, 0.03]	0.12 [-0.47, 0.66]
Action potential max depolarization rate (mV/ms)	461±97 n=26 15 mice	470±103 n=23 14 mice	-0.05	0.36	9 [-46, 63]	0.09 [-0.49, 0.67]
Action potential decay time (ms)	0.16±0.06 n=27 15 mice	0.19±0.10 n=23 14 mice	**0.54**	**0.94**	0.03 [-0.01, 0.08]	0.38 [-0.17, 0.85]
Action potential max repolarization rate (mV/ms)	-406±136 n=26 15 mice	-358±95 n=23 14 mice	0.33	0.46	48 [-13, 113]	0.40 [-0.14, 0.92]
Fast AHP amplitude (mV)	-21±5 n=27 15 mice	-19±4 n=23 14 mice	0.49	0.20	**3 [0.2, 5]**	**0.59 [0.06, 1.22]**
ADP amplitude (mV)	3.3±2.0 n=27 15 mice	4.2±1.8 n=22 14 mice	0.43	-0.34	0.9 [-0.1, 1.9]	0.48 [-0.06, 1.13]
mAHP amplitude (mV)	-21±5 n=26 15 mice	-18±5 n=23 14 mice	0.15	0.44	3 [-0.03, 5]	0.56 [-0.01, 1.31]
mAHP half-width (ms)	44±41 n=24 15 mice	35±20 n=18 14 mice	0.33	-0.25	-9 [-30, 7]	-0.27 [-0.70, 0.39]
Delayed firing motoneurons						
Parameter	Mean±standard deviation		ICC		Bootstrapped Mean difference [95% CI]	Bootstrapped Hedges' *g* [95% CI]
	WT	mSOD1	WT	mSOD1		
Threshold (mV)	-29±5 n=70 28 mice	-28±6 n=115 25 mice	0.03	0.05	1 [-1, 2]	0.14 [-0.14, 0.43]
Action potential amplitude (mV)	63±6 n=73 28 mice	63±8 n=116 25 mice	0.24	0.12	0 [-2, 2]	-0.03 [-0.31, 0.25]
Action potential half-width (ms)	0.22±0.03 n=71	0.21±0.03 n=107	0.12	0.31	0.00 [-0.01, 0.01]	-0.13 [-0.44, 0.18]

	28 mice	23 mice				
Action potential rise time (ms)	0.17±0.03<br>n=70<br>28 mice	0.17±0.02<br>n=107<br>23 mice	0.03	0.22	0.00<br>[-0.01, 0.01]	-0.08<br>[-0.38, 0.22]
Action potential max depolarization rate (mV/ms)	459±66<br>n=70<br>28 mice	457±75<br>n=107<br>23 mice	-<br>0.13	0.27	-2<br>[-23, 19]	-0.03<br>[-0.32, 0.27]
Action potential decay time (ms)	0.15±0.03<br>n=70<br>28 mice	0.15±0.03<br>n=107<br>23 mice	0.37	0.40	0.00<br>[-0.01, 0.004]	-0.14<br>[-0.44, 0.17]
Action potential max repolarization rate (mV/ms)	-386±60<br>n=70<br>28 mice	-384±55<br>n=107<br>23 mice	0.01	0.26	2<br>[-16, 19]	0.03<br>[-0.29, 0.33]
Fast AHP amplitude (mV)	-24±3<br>n=72<br>28 mice	-23±4<br>n=116<br>25 mice	-<br>0.08	0.13	1<br>[-0.3, 2]	0.20<br>[-0.08, 0.48]
ADP amplitude (mV)	4.1±1.9<br>n=72<br>28 mice	4.2±1.9<br>n=114<br>25 mice	0.12	0.41	0.1<br>[-0.5, 0.6]	0.04<br>[-0.26, 0.34]
Medium AHP amplitude (mV)	-23±4<br>n=70<br>28 mice	-23±5<br>n=116<br>25 mice	0.17	0.24	0<br>[-1, 1]	-0.02<br>[-0.30, 0.26]
Medium AHP half-width (ms)	30±13<br>n=69<br>28 mice	26±11<br>n=113<br>25 mice	0.44	0.00	**-4<br>[-8, -1]**	**-0.35<br>[-0.69, -0.05]**

CI - confidence interval; ADP - afterdepolarization; AHP - afterhyperpolarization; for mixed model results when ICC>0.50 see table S24

Table S3 – Mean and standard deviation, intraclass correlation coefficient (ICC), bootstrapped mean difference and Hedges' *g* for recurrent excitatory and inhibitory conductances measured from early and delayed firing motoneurons from oblique slices

Early firing motoneurons						
Parameter	Mean±standard deviation		ICC		Bootstrapped Mean difference [95% CI]	Bootstrapped Hedges' *g* [95% CI]
	WT	mSOD1	WT	mSOD1		
Recurrent excitation (ns)	10±7 n=19 10 mice	9±6 n=14 7 mice	**0.71**	-0.63	2 [-2, 6]	-0.06 [-0.69, 0.65]
Recurrent excitation scaled (%)	31±26 n=19 10 mice	23±22 n=14 7 mice	0.34	**0.53**	-3 [-18, 13]	-0.32 [-1.02, 0.37]
Ventral root-evoked EPSC rise-time	0.46±0.18 n=14 10 mice	0.51±0.10 n=12 7 mice	0.17	**0.67**	0.05 [-0.06, 0.16]	0.33 [-0.41, 1.18]
Ventral root-evoked EPSC decay-time	4.52±3.10 n=14 10 mice	4.74±2.03 n=12 7 mice	**0.91**	**0.88**	0.22 [-1.81, 2.05]	0.08 [-0.60, 1.11]
Recurrent inhibition (nS)	18±17 n=23 15 mice	15±12 n=14 9 mice	-1.18	-0.54	-5 [-15, 5]	-0.21 [-0.78, 0.46]
Recurrent inhibition scaled (%)	44±36 n=23 15 mice	27±19 n=14 9 mice	-0.87	-0.62	-16 [-34, 1]	-0.53 [-1.07, 0.02]
Delayed firing motoneurons						
Parameter	Mean±standard deviation		ICC		Bootstrapped Mean difference [95% CI]	Bootstrapped Hedges' *g* [95% CI]
	WT	mSOD1	WT	mSOD1		
Recurrent excitation (ns)	16±10 n=32 15 mice	16±11 n=38 13 mice	0.17	0.08	1 [-4, 6]	0.07 [-0.41, 0.55]
Recurrent excitation scaled (%)	18±14 n=32 15 mice	17±11 n=38 13 mice	0.34	0.04	-1 [-7, 5]	-0.10 [-0.59, 0.39]
Ventral root-evoked EPSC rise-time	0.52±0.20 n=27 13 mice	0.52±0.11 n=32 11 mice	**0.54**	0.48	0.00 [-0.08, 0.08]	0.00 [-0.54, 0.58]
Ventral root-evoked EPSC decay-time	5.84±1.99 n=26 13 mice	5.04±1.48 n=32 11 mice	**0.52**	0.29	-0.80 [-1.71, 0.10]	-0.46 [0.05, -1.07]
Recurrent inhibition (nS)	78±78 n=83 26 mice	36±32 n=102 23 mice	0.45	0.29	**-42 [-61, -25]**	**-0.74 [-0.98, -0.50]**
Recurrent inhibition scaled (%)	79±78 n=83 26 mice	41±36 n=102 23 mice	0.47	0.42	**-38 [-56, -21]**	**-0.65 [-0.90, -0.40]**

CI - confidence interval; EPSC - excitatory postsynaptic current; for mixed model results when ICC>0.50 see table S24

Table S4 – Mean and standard deviation, intraclass correlation coefficient (ICC), bootstrapped mean difference and Hedges' *g* for Renshaw cell intrinsic properties, ventral root excitation and Bayesian Quantal Analysis (BQA)

Renshaw cells						
Parameter	Mean±standard deviation		ICC		Bootstrapped Mean difference [95% CI]	Bootstrapped Hedges' *g* [95% CI]
	WT	mSOD1	WT	mSOD1		
Cell conductance (nS)	7.8±4.1<br>n=44<br>15 mice	8.3±5.1<br>n=24<br>9 mice	0.06	0.09	0.5<br>[-1.7, 3.0]	0.12<br>[-0.42, 0.66]
Cell capacitance (pF)	99±33<br>n=40<br>15 mice	86±37<br>n=22<br>9 mice	0.37	0.18	-13<br>[-31, 5]	-0.39<br>[-0.96, 0.14]
Ventral root excitation (ns)	9.0±6.0<br>n=44<br>15 mice	7.6±3.3<br>n=23<br>9 mice	0.05	0.11	-1.8<br>[-4.0, 0.3]	-0.27<br>[-0.64, 0.16]
Ventral root excitation scaled (%)	137±100<br>n=44<br>15 mice	117±68<br>n=23<br>9 mice	0.07	0.36	-20<br>[-61, 19]	-0.22<br>[-0.62, 0.25]
Ventral root-evoked EPSC rise-time (ms)	0.58±0.16<br>n=35<br>14 mice	0.77±0.27<br>n=15<br>7 mice	0.27	0.42	**0.19**<br>**[0.05, 0.33]**	**0.97**<br>**[0.30, 1.80]**
Ventral root-evoked EPSC decay-time (ms)	9.49±5.15<br>n=37<br>14 mice	10.09±5.74<br>n=18<br>7 mice	0.27	0.50	0.60<br>[-2.40, 3.69]	0.11<br>[-0.49, 0.73]
BQA quantal size (mV)	0.82±0.55<br>n=13<br>8 mice	0.84±0.65<br>n=11<br>5 mice	-0.44	0.35	0.02<br>[-0.43, 0.50]	0.04<br>[-0.88, 0.87]
BQA number of release sites	31±26<br>n=13<br>8 mice	36±27<br>n=11<br>5 mice	0.04	-0.72	5<br>[-16, 25]	0.19<br>[-0.63, 1.13]
BQA probability of release [Ca^{2+}] = 2mM	0.63±0.29<br>n=13<br>8 mice	0.81±0.20<br>n=11<br>5 mice	**0.66**	-0.23	0.16<br>[-0.03, 0.34]	0.71<br>[-0.04, 1.66]
BQA probability of release [Ca^{2+}] = 1mM	0.21±0.17<br>n=13<br>8 mice	0.31±0.12<br>n=11<br>5 mice	**0.92**	-0.08	0.10<br>[-0.02, 0.20]	0.66<br>[-0.10, 1.97]

CI - confidence interval; EPSC - excitatory postsynaptic current; for mixed model results when ICC>0.50 see table S24

Table S5 – Mean and standard deviation, intraclass correlation coefficient (ICC), bootstrapped mean difference and Hedges' *g* for Bayesian Quantal Analysis (BQA), kinetics of ventral-root evoked EPSCs, aIPSCs amplitude and mIPSCs amplitude, frequency and kinetics obtained from recordings performed from motoneurons from oblique slices

Motoneurons BQA						
Parameter	Mean±standard deviation		ICC		Bootstrapped Mean difference [95% CI]	Bootstrapped Hedges' *g* [95% CI]
	WT	mSOD1	WT	mSOD1		
BQA quantal size (mV)	-0.040±0.017 n=23 11 mice	-0.028±0.012 n=22 10 mice	0.13	-0.04	**0.012 [0.004, 0.020]**	**0.81 [0.26, 1.47]**
BQA number of release sites	258±159 n=23 11 mice	344±275 n=22 10 mice	-0.09	0.33	86 [-42, 218]	0.39 [-0.19, 1.02]
BQA probability of release [Ca^{2+}] = 2mM	0.15±0.11 n=23 11 mice	0.19±0.13 n=22 10 mice	-0.04	0.13	0.03 [-0.04, 0.10]	0.26 [-0.34, 0.86]
BQA probability of release [Ca^{2+}] = 4mM	0.44±0.25 n=23 11 mice	0.53±0.28 n=22 10 mice	-0.29	0.32	0.08 [-0.07, 0.24]	0.32 [-0.26, 0.94]
Ventral root-evoked IPSC rise-time (ms)	0.65±0.17 n=18 8 mice	0.82±0.21 n=23 5 mice	-0.20	0.11	**0.17 [0.06, 0.29]**	**0.90 [0.30, 1.65]**
Ventral root-evoked IPSC decay-time (ms)	4.78±1.25 n=18 18 mice	6.22±1.55 n=22 23 mice	-0.34	-0.25	**1.44 [0.62, 2.31]**	**1.01 [0.44, 1.95]**
aIPSC amplitude (nS)	2.20±1.84 n=1903 19 motoneurons	1.55±1.36 n=2173 21 motoneurons	0.16	0.19	**-0.62 [-1.06, -0.21]***	**-0.39 [-0.64, -0.14]***
aIPSC amplitude scaled (%)	9.47±10.60 n=1903 19 motoneurons	4.12±5.31 n=2173 21 motoneurons	0.37	0.47	**-4.98 [-8.46, -1.73]***	**-0.62 [-0.97, -0.24]***
mIPSC amplitude (nS)	2.80±2.21 n=10743 20 motoneurons	2.04±1.72 n=8745 23 motoneurons	0.29	0.21	**-0.78 [-1.37, -0.22]***	**-0.40 [-0.66, -0.12]***
mIPSC amplitude scaled (%)	6.17±6.08 n=10743 20 motoneurons	4.70±5.74 n=8745 23 motoneurons	0.42	0.35	-1.14 [-3.68, 1.53]*	-0.18 [-0.58, 0.21]*
mIPSC inter-event interval (ms)	55±60 n=10670 20 motoneurons	67±82 n=10399 23 motoneurons	**0.52**	0.47	**31 [7, 61]***	**0.42 [0.14, 0.66]***
mIPSC rise-time	0.40±0.44 n=10582 20 motoneurons	0.46±0.48 n=8832 23 motoneurons	0.06	0.06	0.04 [-0.04, 0.12]*	0.09 [-0.08, 0.26]*
mIPSC decay-time	1.66±0.84 n=10026 20 motoneurons	1.83±0.85 n=8118 23 motoneurons	0.40	0.20	0.06 [-0.35, 0.41]*	0.10 [-0.34, 0.57]*

CI - confidence interval; EPSC - excitatory postsynaptic current; aIPSC - asynchronous inhibitory postsynaptic current; mIPSC - miniature inhibitory postsynaptic current; *2-level hierarchical bootstrapping was used for effect size estimation

Table S6 – Linear mixed model fixed and random-effects variables and partial eta squared (η_p^2) for glycine receptor (GlyR) clusters immunohistochemistry from P21 mice

P21 mice Glycine receptor clusters										
	Mean±SD (total observations)		Fixed effects Estimate [95% CI]		Random effects σ^2 (ICC)					Effect size
Parameter	WT	mSOD 1	WT (intercept)	mSOD1	Pair	Animal	MN	Bouton	Residuals	η_p^2 [90% CI]
GlyR area (μm^2)	1.44E-02±1.94E-02	1.39E-02±1.85E-02	1.49E-02 [1.32E-02 , 1.67E-02]	-6.71E-04 [-1.73E-03, 3.88E-04]	3.19E-06 (0.01)	0.00 (0.00)	0.00 (0.00)	9.60E-06 (0.03)	3.48E-04 (0.96)	0.00 [0.00, 0.00]
GlyR intensity	17.2±7.9	17.7±10.0	18.6 [10.7 , 26.8]	1 [-1.7 , 3.8]	79 (0.81)	4 (0.04)	9 (0.09)	2 (0.02)	3 (0.04)	0.00 [0.00, 0.00]
GlyR perimeter (μm)	0.45±0.37	0.45±0.36	0.46 [0.43 , 0.48]	-0.01 [-0.03 , 0.01	5.85E-04 (0.004)	0.00 (0.00)	4.65E-04 (0.004)	2.49E-03 (0.02)	1.28E-01 (0.97)	0.00 [0.00, 0.00]
GlyR per bouton area (count/μm^2)	10.0±3.30	9.06±3.18	9.77 [7.53, 12.01]	**-0.85 [-1.35, -0.35]**	6.30 (0.54)	0.00 (0.00)	0.71 (0.06)	N/A	4.72 (0.40)	**0.024 [0.01, 0.05]**

CI - confidence interval; ICC - intraclass correlation coefficient;
for WT group: 5 animals, 64 motoneurons, 217 boutons and 4892 clusters; for mSOD1 group: 5 animals, 64 motoneurons, 248 boutons and 5125 clusters. For alternative statistical analysis when $\sigma^2_{Residuals}$>0.50 σ^2_{total} see Table S10.

Table S7 – Linear mixed model fixed and random-effects variables and partial eta squared (η_p^2) for glycine transporter 2 (GlyT2) immunohistochemistry from P21 mice

P21 mice Glycine transporter 2 and motoneuron									
	Mean±SD (total observations)		Fixed effects Estimate [95% CI]		Random effects σ^2 (ICC)				Effect size
Parameter	WT	mSOD1	WT (intercept)	mSOD1	Pair	Animal	MN	Residuals	η_p^2 [90% CI]
GlyT2 area (µm^2)	6.90±5.08	6.89±5.00	6.95 [6.18, 7.73]	0.03 [-1.06, 1.11]	0.00 (0.00)	0.54 (0.01)	1.76 (0.04)	38.32 (0.94)	0.00 [0.00, 0.00]
GlyT2 intensity	22.9±6.98	22.3±9.14	22.7 [17.0, 28.4]	-0.5 [-7.5, 6.6]	10.0 (0.13)	30.8 (0.41)	20.3 (0.27)	13.4 (0.18)	0.00 [0.00, 0.00]
GlyT2 perimeter (µm)	11.60±6.53	11.60±6.59	11.61 [10.96, 12.26]	0.05 [-0.86, 0.97]	0.00 (0.00)	0.32 (0.01)	1.42 (0.03)	41.39 (0.96)	0.00 [0.00, 0.00]
Motoneuron area (µm^2)	726±159	838±188	726 [669, 783]	**113 [33, 193]**	0.00 (0.00)	1863 (0.06)		29043 (0.94)	**0.06 [0.01, 0.14]**
Motoneuron intensity	4.21±1.29	3.82±1.52	4.2 [3.3, 5.2]	-0.4 [-1.6, 0.7]	0.31 (0.14)	0.79 (0.36)		1.09 (0.50)	0.00 [0.00, 0.04]
Motoneuron perimeter (µm)	114±15	120±17	114 [110, 118]	6 [0.2, 12]	0.00 (0.00)	0.00 (0.00)		266 (1.00)	0.03 [0.00, 0.10]

CI - confidence interval; ICC - intraclass correlation coefficient;
for WT group: 5 animals, 64 motoneurons and 217 boutons; for mSOD1 group: 5 animals, 64 motoneurons and 248 boutons. For alternative statistical analysis when $\sigma^2_{Residuals}$>0.50 σ^2_{total} see Table S10.

Table S8 – Linear mixed model fixed and random-effects variables and partial eta squared (η_p^2) for glycine receptor (GlyR) clusters immunohistochemistry from P45 mice

P45 mice Glycine receptor clusters										
	Mean±SD (total observations)		Fixed effects Estimate [95% CI]		Random effects σ^2 (ICC)					Effect size
Parameter	WT	mSOD1	WT (intercept)	mSOD1	Pair	Animal	MN	Bouton	Residuals	η_p^2 [90% CI]
GlyR area (μm^2)	1.37E-02±2.08E-02	1.38E-02±2.13E-02	1.40E-02 [1.19E-02, 1.61E-02]	-3.37E-05 [-1.33E-03, 1.27E-03]	4.62E-06 (0.01)	0.00 (0.00)	6.35E-06 (0.01)	1.29E-05 (0.03)	4.24E-04 (0.95)	0.00 [0.00, 0.00]
GlyR intensity	11.4±3.0	11.7±3.2	11.4 [9.2, 13.7]	0.6 [-0.07, 1.2]	6.5 (0.54)	0.00 (0.00)	3.4 (0.29)	0.6 (0.05)	1.4 (0.12)	0.00 [0.00, 0.00]
GlyR perimeter (μm)	0.44±0.38	0.44±0.38	0.45 [0.41, 0.48]	-1.34E-03 [-0.02, 0.02]	1.44E-03 (0.01)	0.00 (0.00)	1.13E-03 (0.01)	2.73E-03 (0.02)	1.42E-0.1 (0.96)	0.00 [0.00, 0.00]
GlyR per bouton area (count/μm^2)	11.13±2.57	10.15±2.59	11.11 [10.26, 11.97]	**-0.98 [-1.46, -0.49]**	0.78 (0.11)	0.00 (0.00)	0.67 (0.10)		5.38 (0.79)	**0.028 [0.01, 0.06]**

CI - confidence interval; ICC - intraclass correlation coefficient;
for WT group: 5 animals, 72 motoneurons, 258 boutons and 6622 clusters; for mSOD1 group: 5 animals, 73 motoneurons, 275 boutons and 6203 clusters. For alternative statistical analysis when $\sigma^2_{Residuals}$>0.50 σ^2_{total} see Table S10.

Table S9 – Linear mixed model fixed and random-effects variables and partial eta squared (η_p^2) for glycine receptor (GlyR) clusters immunohistochemistry from P45 mice

P45 mice Glycine transporter 2 (GlyT2) and motoneuron									
	Mean±SD (total observations)		Fixed effects Estimate [95% CI]		Random effects σ^2 (ICC)				Effect size
Parameter	WT	mSOD1	WT (intercept)	mSOD1	Pair	Animal	MN	Residuals	η_p^2 [90% CI]
GlyT2 area (μm^2)	5.52±5.12	5.18±5.07	5.54 [5.09, 6.00]	-0.34 [-0.99, 0.30]	0.00 (0.00)	0.11 (0.00)	1.44 (0.06)	24.52 (0.94)	0.00 [0.00, 0.00]
GlyT2 intensity	19.6±7.9	21.0±8.8	19.5 [13.3, 25.6]	1.5 [-0.5, 3.6]	6.9 (0.43)	1.2 (0.07)	4.4 (0.28)	3.6 (0.22)	0.00 [0.00, 0.00]
GlyT2 perimeter (μm)	10.28±5.90	9.81±5.75	10.30 [9.80, 10.80]	-0.48 [-1.06, 0.11]	6.85 (0.43)	1.18 (0.07)	4.41 (0.28)	3.56 (0.22)	0.00 [0.00, 0.00]
Motoneuron area (μm^2)	715±184	681±205	716 [664, 767]	-35 [-98, 29]	822 (0.02)	0.00 (0.00)		37365 (0.98)	0.01 [0.00, 0.05]
Motoneuron intensity	4.04±1.27	4.10±1.69	4.00 [2.8, 5.2]	0.1 [-0.3, 0.4]	1.75 (0.66)	0.01 (0.00)		0.89 (0.33)	0.00 [0.00, 0.03]
Motoneuron perimeter (μm)	113±18	108±18	113 [108, 118]	-5 [-11, 1]	11.1 (0.04)	0.00 (0.00)		305 (0.96)	0.02 [0.00, 0.08]

CI - confidence interval; ICC - intraclass correlation coefficient;
for WT group: 5 animals, 72 motoneurons and 2144 boutons; for mSOD1 group: 5 animals, 73 motoneurons and 2103 boutons. For alternative statistical analysis when $\sigma^2_{Residuals}$>0.50 σ^2_{total} see Table S10.

Table S10 - Mean and standard deviation, intraclass correlation coefficient (ICC), bootstrapped mean difference and Hedges' *g* for glycine receptor (GlyR) immunohistochemistry parameters from P21 and P45 mice

P21 mice Glycine receptor clusters		
Parameter	Bootstrapped<br>Mean difference<br>Estimate [95% CI]	Bootstrapped Hedges' *g*<br>Estimate [95% CI]
GlyR area (μm^2)	-4.48E-04<br>[-1.2E-03, 3.00E-04]	-0.02<br>[-0.06, 0.02]
GlyR perimeter (μm)	6.49E-03<br>[-2.07E-02, 7.54E-03]	-0.02<br>[-0.06, 0.02]
P21 mice Glycine transporter 2 (GlyT2) and motoneuron		
GlyT2 perimeter (μm)	-2.28E-03<br>[-3.94E-01, 3.84E-01]	0.00<br>[-0.06, 0.06]
Motoneuron area (μm^2)	**112<br>[53, 171]**	**0.65<br>[0.30, 1.02]**
Motoneuron intensity	-0.39<br>[-0.87, 0.10]	-0.28<br>[-0.63, 0.07]
Motoneuron perimeter (μm)	**6<br>[0.40, 12]**	**0.36<br>[0.03, 0.73]**
P45 mice Glycine receptor clusters		
GlyR area (μm^2)	5.34E-05<br>[-6.77E-04, 7.77E-04]	0.00<br>[-0.03, 0.04]
GlyR perimeter (μm)	**-0.21<br>[-0.27, -0.15]**	**-0.12<br>[-0.16, -0.09]**
GlyR per bouton area (count/μm^2)	**-0.98<br>[-1.42, -0.54]**	**-0.3<br>[-0.55, -0.21]**
P45 mice Glycine transporter 2 and motoneuron		
GlyT2 area (μm^2)	**-0.33<br>[-0.64, -0.03]**	**-0.07<br>[-0.13, -0.01]**
Motoneuron area (μm^2)	-35<br>[-97, 29]	-0.18<br>[-0.51, 0.15]
Motoneuron perimeter (μm)	-5<br>[-11, 1]	-0.29<br>[-0.62, 0.04]

CI - confidence interval

Table S11 - Mean and standard deviation, intraclass correlation coefficient (ICC), bootstrapped mean difference and Hedges' *g* for all data gathered on dorsal root-evoked monosynaptic Ia excitation, disynaptic Ia/Ib inhibition and post-activation depression

Ventral horn-ablated *in vitro* spinal cord						
All motoneurons						
Parameter	Mean±standard deviation		ICC		Bootstrapped Mean difference [95% CI]	Bootstrapped Hedges' *g* [95% CI]
	WT	mSOD1	WT	mSOD1		
Dorsal root-evoked excitation (nS)	33±28 n=253 25 mice	41±26 n=222 23 mice	0.32	0.40	**8 [3, 13]**	**0.28 [0.10, 0.49]**
Dorsal root-evoked excitation scaled (%)	90±108 n=252 25 mice	99±108 n=222 23 mice	0.17	0.10	10 [-10, 30]	0.09 [-0.09, 0.27]
Dorsal root-evoked EPSC paired-pulse ratio	1.05±0.09 n=215 23 mice	1.01±0.10 n=212 23 mice	0.09	0.23	**-0.04 [-0.06, -0.03]**	**-0.47 [-0.28, -0.67]**
Dorsal root-evoked EPSC rise-time (ms)	0.41±0.10 n=249 25 mice	0.39±0.09 n=218 23 mice	0.28	0.25	**-0.02 [-0.04, -0.001]**	**-0.19 [-0.01, -0.37]**
Dorsal root-evoked EPSC decay-time (ms)	4.47±1.38 n=249 25 mice	4.32±1.12 n=218 23 mice	0.41	0.06	-0.15 [-0.38, -0.08]	-0.12 [-0.30, 0.05]
Post-activation depression (ratio)	0.81±0.09 n=151 16 mice	0.82±0.10 n=169 17 mice	0.12	0.10	0.01 [-0.01, 0.03]	0.14 [-0.08, 0.37]
Dorsal root-evoked inhibition (nS)	76±45 n=126 18 mice	82±51 n=167 18 mice	0.21	0.20	6 [-5, 17]	0.13 [-0.10, 0.36]
Dorsal root-evoked inhibition scaled (%)	190±122 n=124 18 mice	206±249 n=167 18 mice	0.14	0.14	15 [-25, 62]	0.06 [-0.20, 0.22]
Dorsal root-evoked IPSC paired-pulse ratio	1.05±0.07 n=113 17 mice	1.08±0.10 n=158 17 mice	-0.04	0.26	**0.03 [0.01, 0.05]**	**0.30 [0.07, 0.52]**
Dorsal root-evoked IPSC rise-time (ms)	0.53±0.11 n=124 18 mice	0.52±0.11 n=164 18 mice	0.40	0.34	-0.01 [-0.03, 0.02]	-0.09 [-0.33, 0.15]
Dorsal root-evoked IPSC decay-time (ms)	4.73±1.24 n=124 18 mice	0.52±0.11 n=163 18 mice	0.26	0.26	**-0.34 [-0.63, -0.04]**	**-0.26 [-0.51, -0.03]**

CI - confidence interval; ICC - intraclass correlation coefficient; EPSC - excitatory postsynaptic current; IPSC - inhibitory postsynaptic current.

Table S12 - Mean and standard deviation, intraclass correlation coefficient (ICC), bootstrapped mean difference and Hedges' *g* for all data gathered on dorsal root-evoked monosynaptic Ia excitation, disynaptic Ia/Ib inhibition and post-activation depression recorded from motoneurons within L4 segments

Ventral horn-ablated *in vitro* spinal cord						
L4 segment						
Parameter	Mean±standard deviation		ICC		Bootstrapped Mean difference [95% CI]	Bootstrapped Hedges' *g* [95% CI]
	WT	mSOD1	WT	mSOD1		
Dorsal root-evoked excitation (nS)	40±36 n=113 15 mice	36±25 n=80 13 mice	0.35	0.43	-4 [-12, 5]	-0.14 [-0.38, 0.15]
Dorsal root-evoked excitation scaled (%)	114±148 n=113 16 mice	85±67 n=80 13 mice	0.29	0.18	**-29 [-62, -0.4]**	-0.24 [-0.44, 0.01]
Dorsal root-evoked EPSC paired-pulse ratio	1.06±0.08 n=100 15 mice	0.99±0.10 n=78 13 mice	0.18	0.20	**-0.07 [-0.09, -0.04]**	**-0.73 [-1.06, -0.44]**
Dorsal root-evoked EPSC rise-time (ms)	0.40±0.07 n=112 15 mice	0.39±0.10 n=80 13 mice	0.15	0.30	-0.01 [-0.03, 0.02]	-0.07 [-0.39, 0.22]
Dorsal root-evoked EPSC decay-time (ms)	4.22±1.21 n=112 15 mice	4.15±1.14 n=80 13 mice	0.49	0.20	-0.06 [-0.40, 0.28]	-0.05 [-0.34, 0.23]
Post-activation depression (ratio)	0.81±0.08 n=65 12 mice	0.80±0.11 n=61 11 mice	0.10	0.35	-0.01 [-0.04, 0.02]	-0.11 [-0.46, 0.25]
Dorsal root-evoked inhibition (nS)	87±50 n=51 13 mice	80±58 n=58 11 mice	0.20	0.16	-7 [-27, 13]	-0.12 [-0.51, 0.25]
Dorsal root-evoked inhibition scaled (%)	201±127 n=51 13 mice	196±187 n=58 11 mice	0.28	0.09	-5 [-62, 56]	-0.03 [-0.45, 0.31]
Dorsal root-evoked IPSC paired-pulse ratio	1.05±0.07 n=50 13 mice	1.06±0.10 n=56 11 mice	0.29	**0.55**	0.01 [-0.02, 0.04]	0.09 [-0.29, 0.46]
Dorsal root-evoked IPSC rise-time (ms)	0.53±0.10 n=50 13 mice	0.48±0.09 n=58 11 mice	**0.63**	**0.60**	**-0.04 [-0.08, 0.01]**	**-0.46 [-0.86, -0.10]**
Dorsal root-evoked IPSC decay-time (ms)	4.72±1.24 n=50 13 mice	3.99±1.10 n=58 11 mice	0.36	**0.54**	**-0.73 [-1.17, -0.30]**	**-0.62 [-1.00, -0.27]**

CI - confidence interval; ICC - intraclass correlation coefficient; EPSC - excitatory postsynaptic current; IPSC - inhibitory postsynaptic current; for linear mixed model results when ICC>0.50 see table S25

Table S13 - Mean and standard deviation, intraclass correlation coefficient (ICC), bootstrapped mean difference and Hedges' *g* for all data gathered on dorsal root-evoked monosynaptic Ia excitation, disynaptic Ia/Ib inhibition and post-activation depression recorded from motoneurons within L5 segments

Ventral horn-ablated *in vitro* spinal cord						
L5 segment						
Parameter	Mean±standard deviation		ICC		Bootstrapped Mean difference [95% CI]	Bootstrapped Hedges' *g* [95% CI]
	WT	mSOD1	WT	mSOD1		
Dorsal root-evoked excitation (nS)	26±17 n=140 21 mice	43±27 n=142 22 mice	0.34	0.36	**17 [11, 22]**	**0.73 [0.52, 0.95]**
Dorsal root-evoked excitation scaled (%)	70±50 n=139 21 mice	107±125 n=142 22 mice	0.14	0.42	**38 [18, 62]**	**0.39 [0.26, 0.58]**
Dorsal root-evoked EPSC paired-pulse ratio	1.05±0.09 n=115 19 mice	1.02±0.10 n=134 22 mice	0.06	0.22	**-0.03 [-0.05, -0.01]**	**-0.32 [-0.57, -0.06]**
Dorsal root-evoked EPSC rise-time (ms)	0.43±0.12 n=137 21 mice	0.40±0.09 n=138 22 mice	0.38	0.31	**-0.03 [-0.06, -0.01]**	**-0.28 [-0.52, -0.05]**
Dorsal root-evoked EPSC decay-time (ms)	4.67±1.48 n=137 21 mice	4.41±1.10 n=138 22 mice	0.44	0.22	-0.26 [-0.57, 0.04]	-0.20 [-0.44, 0.23]
Post-activation depression (ratio)	0.80±0.10 n=86 15 mice	0.83±0.09 n=107 16 mice	0.28	0.04	**0.27 [0.00, 0.05]**	**0.29 [0.01, 0.59]**
Dorsal root-evoked inhibition (nS)	68±39 n=75 15 mice	83±47 n=109 15 mice	0.16	0.27	**15 [2, 27]**	**0.33 [0.05, 0.62]**
Dorsal root-evoked inhibition scaled (%)	183±119 n=73 15 mice	210±278 n=109 15 mice	0.14	**0.86**	28 [-24, 92]	0.12 [-0.19, 0.30]
Dorsal root-evoked IPSC paired-pulse ratio	1.05±0.07 n=63 14 mice	1.09±0.10 n=102 14 mice	-0.13	0.31	**0.04 [0.01, 0.06]**	**0.41 [0.13, 0.68]**
Dorsal root-evoked IPSC rise-time (ms)	0.53±0.11 n=74 15 mice	0.54±0.11 n=106 15 mice	0.32	0.19	0.01 [-0.02, 0.04]	0.09 [-0.21, 0.39]
Dorsal root-evoked IPSC decay-time (ms)	4.74±1.25 n=74 15 mice	4.62±1.43 n=105 15 mice	0.31	0.29	-0.13 [-0.54, 0.26]	-0.10 [-0.41, 0.19]

CI - confidence interval; ICC - intraclass correlation coefficient; EPSC - excitatory postsynaptic current; IPSC - inhibitory postsynaptic current; for linear mixed model results when ICC>0.50 see table S25

Table S14 - Mean and standard deviation, intraclass correlation coefficient (ICC), bootstrapped mean difference and Hedges' *g* for all data gathered on L4 dorsal root-evoked monosynaptic Ia excitation, disynaptic Ia/Ib inhibition and post-activation depression recorded from motoneurons within L4 segments

Ventral horn-ablated *in vitro* spinal cord						
L4 segment and L4 dorsal root stimulation						
Parameter	Mean±standard deviation		ICC		Bootstrapped Mean difference [95% CI]	Bootstrapped Hedges' *g* [95% CI]
	WT	mSOD1	WT	mSOD1		
Dorsal root-evoked excitation (nS)	44±36<br>n=57<br>15 mice	44±26<br>n=39<br>11 mice	0.33	0.44	0<br>[-13, 12]	-0.01<br>[-0.36, 0.41]
Dorsal root-evoked excitation scaled (%)	119±109<br>n=57<br>15 mice	105±76<br>n=39<br>11 mice	0.35	0.02	-14<br>[-51, 22]	-0.14<br>[-0.49, 0.25]
Dorsal root-evoked EPSC paired-pulse ratio	1.07±0.07<br>n=49<br>15 mice	1.00±0.09<br>n=38<br>11 mice	0.23	0.25	**0.07**<br>**[-0.10, -0.03]**	-0.84<br>[-1.34, 0.42]
Dorsal root-evoked EPSC rise-time (ms)	0.40±0.08<br>n=57<br>15 mice	0.38±0.10<br>n=39<br>11 mice	0.04	0.26	-0.02<br>[-0.06, 0.01]	-0.27<br>[-0.76, 0.15]
Dorsal root-evoked EPSC decay-time (ms)	4.45±1.25<br>n=57<br>15 mice	4.27±1.24<br>n=39<br>11 mice	0.45	0.06	-0.18<br>[-0.67, 0.34]	-0.15<br>[-0.57, 0.27]
Post-activation depression (ratio)	0.82±0.06<br>n=32<br>12 mice	0.82±0.10<br>n=28<br>10 mice	0.13	**0.65**	0.00<br>[-0.05, 0.04]	-0.05<br>[-0.57, 0.51]
Dorsal root-evoked inhibition (nS)	85±54<br>n=29<br>13 mice	103±63<br>n=31<br>10 mice	0.00	0.34	18<br>[-11, 47]	0.31<br>[-0.19, 0.86]
Dorsal root-evoked inhibition scaled (%)	207±139<br>n=29<br>13 mice	255±216<br>n=31<br>10 mice	-0.02	0.23	49<br>[-39, 143]	0.27<br>[-0.26, 0.71]
Dorsal root-evoked IPSC paired-pulse ratio	1.05±0.06<br>n=29<br>13 mice	1.04±0.09<br>n=30<br>10 mice	0.43	**0.68**	-0.01<br>[-0.04, 0.04]	-0.05<br>[-0.64, 0.42]
Dorsal root-evoked IPSC rise-time (ms)	0.53±0.12<br>n=28<br>13 mice	0.47±0.09<br>n=31<br>10 mice	**0.63**	**0.64**	**-0.06**<br>**[-0.11, -0.01]**	**-0.55**<br>**[-1.07, -0.06]**
Dorsal root-evoked IPSC decay-time (ms)	4.83±1.20<br>n=28<br>13 mice	4.30±1.17<br>n=31<br>10 mice	0.04	0.45	-0.54<br>[-1.13, 0.07]	-0.45<br>[-0.99, 0.05]

CI - confidence interval; ICC - intraclass correlation coefficient; EPSC - excitatory postsynaptic current; IPSC - inhibitory postsynaptic current; for linear mixed model results when ICC>0.50 see table S25

Table S15 - Mean and standard deviation, intraclass correlation coefficient (ICC), bootstrapped mean difference and Hedges' *g* for all data gathered on L5 dorsal root-evoked monosynaptic Ia excitation, disynaptic Ia/Ib inhibition and post-activation depression recorded from motoneurons within L4 segments

Ventral horn-ablated *in vitro* spinal cord						
L4 segment and L5 dorsal root stimulation						
Parameter	Mean±standard deviation		ICC		Bootstrapped Mean difference [95% CI]	Bootstrapped Hedges' *g* [95% CI]
	WT	mSOD1	WT	mSOD1		
Dorsal root-evoked excitation (nS)	36±36 n=56 14 mice	29±22 n=41 13 mice	0.28	**0.56**	-8 [-20, 3]	-0.27 [-0.59, 0.11]
Dorsal root-evoked excitation scaled (%)	108±180 n=56 14 mice	65±50 n=41 13 mice	0.26	0.40	**-44 [-99, -1]**	**-0.32 [-0.54, -0.02]**
Dorsal root-evoked EPSC paired-pulse ratio	1.04±0.09 n=51 14 mice	0.98±0.11 n=40 13 mice	0.09	0.29	**-0.06 [-0.11, -0.02]**	**-0.66 [-1.11, -0.25]**
Dorsal root-evoked EPSC rise-time (ms)	0.39±0.06 n=55 14 mice	0.40±0.10 n=41 13 mice	0.25	**0.54**	0.01 [-0.02, 0.05]	0.15 [-0.28, 0.58]
Dorsal root-evoked EPSC decay-time (ms)	3.98±1.12 n=55 14 mice	4.05±1.05 n=41 13 mice	0.50	0.36	0.07 [-0.37, 0.50]	0.06 [-0.34, 0.48]
Post-activation depression (ratio)	0.80±0.09 n=33 10 mice	0.78±0.12 n=33 10 mice	**0.57**	0.28	-0.01 [-0.06, 0.04]	-0.13 [-0.61, 0.37]
Dorsal root-evoked inhibition (nS)	90±47 n=22 10 mice	54±39 n=27 9 mice	0.20	0.40	**-36 [-59, -12]**	**-0.83 [-1.51, -0.30]**
Dorsal root-evoked inhibition scaled (%)	194±111 n=22 10 mice	129±116 n=27 9 mice	0.50	-0.11	**-65 [-128, -3]**	**-0.57 [-1.38, -0.03]**
Dorsal root-evoked IPSC paired-pulse ratio	1.05±0.08 n=21 10 mice	1.07±0.11 n=26 9 mice	0.36	**0.53**	0.02 [-0.03, 0.07]	0.23 [-0.32, 0.84]
Dorsal root-evoked IPSC rise-time (ms)	0.52±0.07 n=22 10 mice	0.49±0.10 n=27 9 mice	0.43	**0.52**	-0.03 [-0.07, 0.02]	-0.32 [-0.98, 0.21]
Dorsal root-evoked IPSC decay-time (ms)	4.58±1.31 n=22 10 mice	3.64±0.92 n=27 9 mice	0.27	**0.62**	**-0.93 [-1.60, -0.34]**	**-0.84 [-1.34, -0.37]**

CI - confidence interval; ICC - intraclass correlation coefficient; EPSC - excitatory postsynaptic current; IPSC - inhibitory postsynaptic current; for linear mixed model results when ICC>0.50 see table S25

Table S16 - Mean and standard deviation, intraclass correlation coefficient (ICC), bootstrapped mean difference and Hedges' *g* for all data gathered on L4 dorsal root-evoked monosynaptic Ia excitation, disynaptic Ia/Ib inhibition and post-activation depression recorded from motoneurons within L5 segments

Ventral horn-ablated *in vitro* spinal cord						
L5 segment and L4 dorsal root stimulation						
Parameter	Mean±standard deviation		ICC		Bootstrapped Mean difference [95% CI]	Bootstrapped Hedges' *g* [95% CI]
	WT	mSOD1	WT	mSOD1		
Dorsal root-evoked excitation (nS)	30±19 n=63 17 mice	42±26 n=66 19 mice	**0.55**	0.19	**12 [4, 20]**	**0.54 [0.22, 0.87]**
Dorsal root-evoked excitation scaled (%)	76±49 n=63 17 mice	108±143 n=66 19 mice	0.30	0.37	**32 [2, 73]**	**0.29 [0.04, 0.57]**
Dorsal root-evoked EPSC paired-pulse ratio	1.06±0.08 n=60 17 mice	1.02±0.10 n=62 19 mice	0.19	0.15	**-0.05 [-0.08, -0.01]**	**-0.51 [-0.91, -0.14]**
Dorsal root-evoked EPSC rise-time (ms)	0.46±0.14 n=63 17 mice	0.41±0.09 n=64 19 mice	0.46	0.10	**-0.05 [-0.09, -0.01]**	**-0.39 [-0.75, -0.05]**
Dorsal root-evoked EPSC decay-time (ms)	4.82±1.68 n=63 17 mice	4.46±1.04 n=64 19 mice	0.45	0.29	-0.36 [-0.85, 0.10]	-0.26 [-0.61, 0.08]
Post-activation depression (ratio)	0.82±0.11 n=35 11 mice	0.81±0.07 n=53 14 mice	0.17	0.03	-0.02 [-0.06, 0.02]	-0.18 [-0.61, 0.29]
Dorsal root-evoked inhibition (nS)	72±40 n=39 11 mice	80±47 n=49 13 mice	0.17	0.36	8 [-10, 26]	0.18 [-0.24, 0.59]
Dorsal root-evoked inhibition scaled (%)	195±106 n=39 11 mice	232±392 n=49 13 mice	-0.07	**0.94**	37 [-51, 173]	0.12 [-0.50, 0.35]
Dorsal root-evoked IPSC paired-pulse ratio	1.05±0.07 n=34 11 mice	1.09±0.08 n=47 13 mice	0.19	0.26	**0.04 [0.01, 0.07]**	**0.50 [0.10, 0.90]**
Dorsal root-evoked IPSC rise-time (ms)	0.56±0.13 n=39 11 mice	0.57±0.12 n=49 13 mice	0.47	-0.06	0.02 [-0.04, 0.07]	0.13 [-0.32, 0.55]
Dorsal root-evoked IPSC decay-time (ms)	4.63±1.39 n=39 11 mice	4.54±1.39 n=49 13 mice	0.26	0.09	-0.09 [-0.65, 0.50]	-0.07 [-0.54, 0.33]

CI - confidence interval; ICC - intraclass correlation coefficient; EPSC - excitatory postsynaptic current; IPSC - inhibitory postsynaptic current; for linear mixed model results when ICC>0.50 see table S25

Table S17 – Mean and standard deviation, intraclass correlation coefficient (ICC), bootstrapped mean difference and Hedges' *g* for all data gathered on L5 dorsal root-evoked monosynaptic Ia excitation, disynaptic Ia/Ib inhibition and post-activation depression recorded from motoneurons within L5 segments

Ventral horn-ablated *in vitro* spinal cord						
L5 segment and L5 dorsal root stimulation						
Parameter	Mean±standard deviation		ICC		Bootstrapped Mean difference Estimate [95% CI]	Bootstrapped Hedges' *g* Estimate [95% CI]
	WT	mSOD1	WT	mSOD1		
Dorsal root-evoked excitation (nS)	24±16<br>n=77<br>19 mice	44±27<br>n=76<br>20 mice	0.25	0.47	**20<br>[13, 27]**	**0.90<br>[0.61, 1.20]**
Dorsal root-evoked excitation scaled (%)	65±50<br>n=76<br>19 mice	107±107<br>n=76<br>20 mice	0.07	0.33	**42<br>[18, 72]**	**0.50<br>[0.29, 0.81]**
Dorsal root-evoked EPSC paired-pulse ratio	1.04±0.11<br>n=55<br>17 mice	1.02±0.10<br>n=72<br>20 mice	0.20	0.19	-0.01<br>[-0.05, 0.02]	-0.15<br>[-0.49, 0.21]
Dorsal root-evoked EPSC rise-time (ms)	0.40±0.10<br>n=74<br>19 mice	0.38±0.09<br>n=74<br>20 mice	0.28	**0.52**	-0.02<br>[-0.05, 0.01]	-0.18<br>[-0.50, 0.15]
Dorsal root-evoked EPSC decay-time (ms)	4.55±1.29<br>n=74<br>19 mice	4.37±1.17<br>n=74<br>20 mice	0.42	0.28	-0.18<br>[-0.57, 0.21]	-0.15<br>[-0.49, 0.17]
Post-activation depression (ratio)	0.79±0.09<br>n=51<br>14 mice	0.85±0.10<br>n=54<br>14 mice	0.23	-0.06	**0.06<br>[0.03, 0.10]**	**0.68<br>[0.31, 1.08]**
Dorsal root-evoked inhibition (nS)	64±38<br>n=36<br>15 mice	85±47<br>n=60<br>12 mice	0.28	0.25	**21<br>[4, 38]**	**0.48<br>[0.09, 0.89]**
Dorsal root-evoked inhibition scaled (%)	169±133<br>n=34<br>15 mice	193±123<br>n=60<br>12 mice	0.19	0.06	25<br>[-31, 77]	0.19<br>[-0.24, 0.66]
Dorsal root-evoked IPSC paired-pulse ratio	1.06±0.06<br>n=29<br>14 mice	1.09±0.11<br>n=55<br>12 mice	-0.30	0.31	0.03<br>[-0.01, 0.07]	0.33<br>[-0.05, 0.69]
Dorsal root-evoked IPSC rise-time (ms)	0.50±0.08<br>n=35<br>15 mice	0.51±0.08<br>n=57<br>12 mice	0.18	**0.61**	0.03<br>[-0.01, 0.07]	0.15<br>[-0.28, 0.57]
Dorsal root-evoked IPSC decay-time (ms)	4.86±1.09<br>n=35<br>15 mice	4.69±1.47<br>n=56<br>12 mice	**0.51**	0.32	-0.20<br>[-0.71, 0.33]	-0.15<br>[-0.58, 0.24]

CI - confidence interval; ICC - intraclass correlation coefficient; EPSC - excitatory postsynaptic current; IPSC - inhibitory postsynaptic current

Table S18 – Mean and standard deviation, bootstrapped mean difference and Hedges' *g* for motoneuron resting conductance and capacitance and latency and jitter obtained from dorsal root-evoked monosynaptic EPSCs and disynaptic IPSCs from *in vitro* partially-ventral horn ablated spinal cord preparations

Parameter	Mean±standard deviation		Bootstrapped Mean difference Estimate [95% CI]	Bootstrapped Hedges' *g* Estimate [95% CI]
	WT	mSOD1		
Motoneuron conductance (nS)	46±28<br>n=247<br>35 mice	47±22<br>n=198<br>30 mice	0.9<br>[-4, 5]	0.04<br>[-0.14, 0.23]
Motoneuron capacitance (pF)	260±79<br>n=243<br>35 mice	271±86<br>n=199<br>30 mice	11<br>[-4, 27]	0.14<br>[-0.05, 0.33]
	EPSC	IPSC		
WT latency (ms)	0.80±0.11<br>n=68<br>16 mice	0.85±0.12<br>n=68<br>16 mice	**0.05**<br>**[0.03, 0.07]**	**0.40**<br>**[0.24, 0.59]**
WT jitter (ms)	0.014±0.006<br>n=68<br>16 mice	0.036±0.020<br>n=68<br>16 mice	**0.019**<br>**[0.015, 0.024]**	**1.28**<br>**[1.05, 1.61]**
mSOD1 latency (ms)	0.79±0.12<br>n=125<br>16 mice	0.86±0.14<br>n=125<br>16 mice	**0.07**<br>**[0.06, 0.08]**	**0.55**<br>**[0.41, 0.71]**
mSOD1 jitter (ms)	0.013±0.004<br>n=125<br>16 mice	0.029±0.017<br>n=125<br>16 mice	**0.015**<br>**[0.013, 0.019]**	**1.32**<br>**[1.07, 1.67]**

CI - confidence interval; EPSC - excitatory postsynaptic current; IPSC - inhibitory postsynaptic current

Table S19 - Mean and standard deviation, intraclass correlation coefficient (ICC), bootstrapped mean difference and Hedges' *g* for Bayesian Quantal Analysis (BQA) performed on monosynaptic Ia excitation

In vitro ventral horn-ablated spinal cord						
Bayesian Quantal Analysis						
Parameter	Mean±standard deviation		ICC		Bootstrapped Mean difference Estimate [95% CI]	Bootstrapped Hedges' *g* Estimate [95% CI]
	WT	mSOD1	WT	mSOD1		
Quantal size (nS)	0.38±0.14<br>n=36<br>13 mice	0.37±0.19<br>n=27<br>9 mice	-0.10	0.22	-0.01<br>[-0.09, 0.07]	-0.09<br>[-0.64, 0.44]
Quantal size scaled (%)	1.46±1.15<br>n=36<br>13 mice	1.58±1.46<br>n=27<br>9 mice	**0.52**	0.09	0.11<br>[-0.52, 0.80]	0.09<br>[-0.46, 0.58]
Number of release sites	245±123<br>n=37<br>14 mice	201±132<br>n=27<br>9 mice	-0.03	0.13	-44<br>[-106, 18]	-0.35<br>[-0.87, 0.15]
Probability of release [Ca^{2+}] = 2mM	0.23±0.10<br>n=37<br>14 mice	0.33±0.15<br>n=27<br>9 mice	-0.05	0.06	**0.11<br>[0.05, 0.17]**	**0.83<br>[0.32, 1.42]**
Probability of release [Ca^{2+}] = 4mM	0.48±0.17<br>n=36<br>14 mice	0.63±0.23<br>n=27<br>9 mice	0.05	-0.22	**0.16<br>[0.06, 0.26]**	**0.79<br>[0.31, 1.40]**

CI - confidence interval; ICC - intraclass correlation coefficient

Table S20 - Mean and standard deviation, bootstrapped mean difference and Hedges' *g* for recurrent inhibition studied through electromyography (EMG) recordings

	Parameter	Mean±standard deviation		Bootstrapped Mean difference Estimate [95% CI]	Bootstrapped Hedges' *g* Estimate [95% CI]
Age range	Conditioning interval	WT	mSOD1		
P18-23	2 ms (% control H-reflex)	101±28 n=13 mice	107±27 n=11 mice	6 [-15, 27]	0.22 [-0.60, 1.08]
	5 ms (% control H-reflex)	102±37 n=15 mice	101±20 n=12 mice	-1 [-22, 19]	-0.04 [-0.69, 0.90]
	10 ms (% control H-reflex)	89±14 n=14 mice	105±18 n=10 mice	**16 [4, 29]**	**1.02 [0.27, 1.98]**
	20 ms (% control H-reflex)	82±25 n=14 mice	99±8 n=12 mice	**17 [3, 30]**	**0.85 [0.21, 1.70]**
	30 ms (% control H-reflex)	82±13 n=14 mice	99±11 n=11 mice	**17 [8, 26]**	**1.41 [0.71, 2.58]**
	50 ms (% control H-reflex)	80±22 n=14 mice	98±11 n=10 mice	**17 [5, 31]**	**0.94 [0.42, 1.63]**
	100 ms (% control H-reflex)	89±16 n=14 mice	98±16 n=8 mice	9 [-3, 22]	0.60 [-0.25, 1.53]
P57-67	2 ms (% control H-reflex)	102±30 n=11 mice	84±22 n=11 mice	-18 [-41, 2]	-0.67 [-1.56, 0.11]
	5 ms (% control H-reflex)	85±23 n=11 mice	83±23 n=11 mice	-2 [-20, 16]	-0.09 [-0.97, 0.78]
	10 ms (% control H-reflex)	82±18 n=11 mice	85±23 n=10 mice	3 [-14, 20]	0.14 [-0.81, 1.05]
	20 ms (% control H-reflex)	94±17 n=11 mice	84±19 n=10 mice	-10 [-26, 3]	-0.58 [-1.39, 0.27]
	30 ms (% control H-reflex)	96±16 n=11 mice	96±8 n=10 mice	0 [-10, 10]	0.01 [-1.00, 0.83]
	50 ms (% control H-reflex)	92±13 n=11 mice	102±4 n=10 mice	**9 [1, 16]**	**0.94 [0.10, 2.75]**
P87-108	2 ms (% control H-reflex)	101±17 n=10 mice	81±24 n=11 mice	**-20 [-38, -4]**	**-0.93 [-1.88, -0.20]**
	5 ms (% control H-reflex)	96±24 n=10 mice	85±18 n=11 mice	-10 [-28, 6]	-0.49 [-1.37, 0.38]
	10 ms (% control H-reflex)	87±23 n=9 mice	78±29 n=11 mice	-10 [-31, 12]	-0.36 [-1.44, 0.47]
	20 ms (% control H-reflex)	88±21 n=10 mice	98±20 n=11 mice	10 [-6, 28]	0.50 [-0.37, 1.32]
	30 ms (% control H-reflex)	103±19 n=9 mice	93±19 n=11 mice	-9 [-26, 5]	-0.49 [-1.29, 0.45]
	50 ms (% control H-reflex)	93±16 n=9 mice	97±10 n=11 mice	3 [-7, 15]	0.24 [-0.91, 1.05]

CI - confidence interval; ICC - intraclass correlation coefficient

Table S21 - Mean and standard deviation, bootstrapped mean difference and Hedges' *g* for post-activation depression studied through EMG recordings

	Parameter	Mean±standard deviation		Bootstrapped Mean difference Estimate [95% CI]	Bootstrapped Hedges' *g* Estimate [95% CI]
Age range	Conditioning interval	WT	mSOD1		
P18-23	50 ms (% control H-reflex)	37±16 n=15 mice	47±22 n=9 mice	9 [-6, 26]	0.51 [-0.38, 1.37]
	100 ms (% control H-reflex)	46±20 n=15 mice	45±24 n=8 mice	0 [-18, 19]	-0.02 [-1.05, 0.87]
	200 ms (% control H-reflex)	47±15 n=14 mice	54±23 n=10 mice	7 [-9, 23]	0.36 [-0.55, 1.29]
	500 ms (% control H-reflex)	54±19 n=14 mice	64±22 n=11 mice	10 [-5, 26]	0.51 [-0.27, 1.40]
	1 s (% control H-reflex)	70±23 n=14 mice	64±27 n=10 mice	-6 [-25, 13]	-0.26 [-1.24, 0.58]
	5 s (% control H-reflex)	83±15 n=15 mice	97±13 n=10 mice	**15 [4, 25]**	**1.01 [0.25, 2.17]**
	10 s (% control H-reflex)	97±7 n=15 mice	102±11 n=10 mice	4 [-3, 12]	0.52 [-0.35, 1.41]
	20 s (% control H-reflex)	102±5 n=13 mice	107±6 n=11 mice	**6 [1, 10]**	**0.99 [0.19, 2.29]**
P57-67	50 ms (% control H-reflex)	50±19 n=11 mice	52±20 n=6 mice	2 [-16, 20]	0.09 [-0.98, 1.18]
	100 ms (% control H-reflex)	54±20 n=11 mice	56±15 n=7 mice	2 [-13, 17]	0.13 [-0.86, 1.06]
	200 ms (% control H-reflex)	55±18 n=11 mice	63±16 n=7 mice	8 [-7, 24]	0.48 [-0.45, 1.49]
	500 ms (% control H-reflex)	62±15 n=11 mice	75±13 n=7 mice	**13 [1, 25]**	**0.92 [0.15, 1.91]**
	1 s (% control H-reflex)	70±16 n=11 mice	80±14 n=7 mice	10 [-4, 22]	0.63 [-0.24, 1.82]
	5 s (% control H-reflex)	88±10 n=11 mice	94±12 n=7 mice	6 [-4, 16]	0.59 [-0.37, 1.82]
	10 s (% control H-reflex)	94±10 n=11 mice	100±12 n=7 mice	6 [-4, 16]	0.55 [-0.41, 1.92]
	20 s (% control H-reflex)	103±5 n=11 mice	102±6 n=6 mice	-1 [-6, 4]	-0.20 [-1.39, 0.90]
P87-108	50 ms (% control H-reflex)	49±20 n=9 mice	51±21 n=11 mice	1 [-16, 18]	0.05 [-0.93, 0.93]
	100 ms (% control H-reflex)	54±21 n=9 mice	62±19 n=13 mice	9 [-7, 25]	0.45 [-0.41, 1.65]
	200 ms (% control H-reflex)	53±18 n=9 mice	70±22 n=12 mice	**17 [0.1, 33]**	**0.81 [0.03, 2.11]**
	500 ms (% control H-reflex)	70±16 n=9 mice	79±19 n=11 mice	9 [-6, 23]	0.52 [-0.33, 1.68]
	1 s (% control H-reflex)	72±16 n=9 mice	95±15 n=13 mice	**23 [10, 36]**	**1.46 [0.73, 2.63]**
	5 s (% control H-reflex)	92±13 n=9 mice	97±11 n=13 mice	5 [-5, 14]	0.40 [-0.46, 1.47]
	10 s (% control H-reflex)	99±9 n=9 mice	97±13 n=13 mice	-2 [-11, 7]	-0.14 [-0.92, 0.81]
	20 s (% control H-reflex)	101±4 n=9 mice	102±4 n=13 mice	1 [-2, 4]	0.17 [-0.75, 1.10]

CI - confidence interval

Table S22 - Mean and standard deviation, bootstrapped mean difference and Hedges' *g* for Mmax, Hmax and H_{max}/M_{max} (ratio) for Quadriceps and Tibialis Anterior muscles studied through EMG recordings

	Parameter	Mean±standard deviation		Bootstrapped Mean difference Estimate [95% CI]	Bootstrapped Hedges' *g* Estimate [95% CI]
Age range	Conditioning interval	WT	mSOD1		
P18-23	Quadriceps M_{max} (mV)	10±4 n=18 mice	8±3 n=18 mice	-1 [-3, 2]	-0.35 [-1.08, 0.29]
	Tibialis Anterior M_{max} (mV)	21±6 n=21 mice	17±7 n=19 mice	**-4 [-8, -0.5]**	**-0.71 [-1.48, -0.10]**
	Tibialis Anterior H_{max} (mV)	4±2 n=20 mice	3±2 n=17 mice	**-2 [-3, -0.1]**	-0.44 [-1.19, 0.21]
	Tibialis Anterior H_{max}/M_{max} (ratio)	0.21±0.11 n=20 mice	0.23±0.17 n=17 mice	0.02 [-0.07, 0.12]	0.14 [-0.61, 0.73]
P57-67	Quadriceps M_{max} (mV)	13±8 n=14 mice	7±4 n=15 mice	**-6 [-10, -1]**	**-0.93 [-1.73, -0.32]**
	Tibialis Anterior M_{max} (mV)	25±9 n=14 mice	13±5 n=15 mice	**-11 [-18, -5]**	**-1.62 [-2.73, -1.08]**
	Tibialis Anterior H_{max} (mV)	3±1 n=11 mice	3±1 n=10 mice	0 [-1, 1]	-0.27 [-1.27, 0.58]
	Tibialis Anterior H_{max}/M_{max} (ratio)	0.11±0.05 n=11 mice	0.21±0.05 n=10 mice	**0.11 [0.06, 0.16]**	**1.81 [1.00, 3.39]**
P87-108	Quadriceps M_{max} (mV)	11±6 n=14 mice	7±5 n=15 mice	**-5 [-9, -0.2]**	**-0.77 [-1.91, -0.02]**
	Tibialis Anterior M_{max} (mV)	19±6 n=14 mice	10±2 n=16 mice	**-10 [-13, -6]**	**-1.83 [-2.71, -1.33]**
	Tibialis Anterior H_{max} (mV)	3±2 n=12 mice	3±1 n=13 mice	0 [-1, 1]	-0.10 [-0.88, 0.82]
	Tibialis Anterior H_{max}/M_{max} (ratio)	0.17±0.12 n=12 mice	0.30±0.15 n=13 mice	**0.13 [0.02, 0.23]**	**0.92 [0.14, 2.02]**

CI - confidence interval

Table S23 - Mean and standard deviation, intraclass correlation coefficient (ICC), bootstrapped mean difference and Hedges' *g* for recurrent inhibition estimated from *in vivo* and *in vitro* dorsal horn-ablated motoneuron recordings

Ventral horn-ablated *in vitro* spinal cord						
In vivo recordings						
Parameter	Mean±standard deviation		ICC		Bootstrapped Mean difference Estimate [95% CI]	Bootstrapped Hedges' *g* Estimate [95% CI]
	WT	mSOD1	WT	mSOD1		
Recurrent inhibition (nS)	29±23<br>n=22<br>6 mice	26±37<br>n=18<br>7 mice	0.38	**0.64**	-4<br>[-22, 16]	-0.12<br>[-0.98, 0.49]
Recurrent inhibition scaled (%)	10±11<br>n=22<br>6 mice	7±9<br>n=18<br>7 mice	0.25	**0.55**	-3<br>[-9, 3]	-0.27<br>[-0.85, 0.37]
Dorsal horn-ablated *in vitro* spinal cord						
Early firing motoneurons						
Parameter	Mean±standard deviation		ICC		Bootstrapped Mean difference Estimate [95% CI]	Bootstrapped Hedges' *g* Estimate [95% CI]
	WT	mSOD1	WT	mSOD1		
Recurrent inhibition (nS)	27±25<br>n=20<br>7 mice	9±9<br>n=14<br>6 mice	0.19	0.07	**-18<br>[-30, -7]**	**-0.91<br>[-1.49, -0.41]**
Recurrent inhibition scaled (%)	61±68<br>n=20<br>7 mice	31±44<br>n=14<br>6 mice	-0.02	-0.08	-29<br>[-66, 8]	-0.42<br>[-0.97, 0.21]
Delayed firing motoneurons						
Parameter	Mean±standard deviation		ICC		Bootstrapped Mean difference Estimate [95% CI]	Bootstrapped Hedges g Estimate [95% CI]
	WT	mSOD1	WT	mSOD1		
Recurrent inhibition (nS)	63±50<br>n=126<br>11 mice	45±33<br>n=142<br>9 mice	0.27	0.35	**-18<br>[-29, -8]**	**-0.44<br>[-0.65, -0.21]**
Recurrent inhibition scaled (%)	89±86<br>n=126<br>11 mice	53±35<br>n=142<br>9 mice	0.43	0.24	**-36<br>[-53, -20]**	**-0.55<br>[-0.74, -0.36]**

CI - confidence interval; ICC - intraclass correlation coefficient; for linear mixed model results when ICC>0.50 see table S26

Table S24 – Linear mixed model fixed and random-effects variables and partial eta squared (η_p^2) for electrophysiological parameters obtained from early and delayed firing motoneurons and Renshaw cells recorded from oblique spinal cord slices

Oblique spinal cord slices						
		Fixed effects Estimate [95% CI]		Random effects σ^2 (ICC)		Effect size
	Parameter	WT (intercept)	mSOD1	Animal	Residuals	η_p^2 [90% CI]
Early firing motoneurons	Conductance (nS)	52 [39, 65]	4 [-16, 24]	198 (0.15)	1102 (0.85)	0.00 [0.00, 0.06]
	Membrane potential (mV)	-62 [-64, -60]	0 [-2, 3]	5.0 (0.34)	9.8 (0.66)	0.00 [0.00, 0.04]
	1-2nd spike interval (Hz)	391 [268, 514]	35 [-120, 191]	11131 (0.47)	12746 (0.53)	0.00 [0.00, 0.16]
	Action potential half-width (ms)	0.23 [0.19, 0.28]	0.03 [-0.04, 0.10]	7.50E-03 (0.84)	1.41E-03 (0.16)	0.01 [0.00, 0.12]
	Action potential rise time (ms)	0.18 [0.16, 0.20]	0.00 [-0.02, 0.03]	7.50E-03 (0.88)	1.07E-03 (0.12)	0.01 [0.00, 0.07]
	Action potential decay time (ms)	0.16 [0.11, 0.21]	0.04 [-0.04, 0.11]	8.15E-03 (0.86)	1.38E-03 (0.14)	0.02 [0.00, 0.14]
	Recurrent excitation (ns)	10 [6, 13]	0 [-6, 6]	17 (0.38)	28 (0.62)	0.00 [0.00, 0.00]
	Recurrent excitation scaled (%)	32 [18, 46]	-10 [-31, 12]	242 (0.40)	355 (0.60)	0.03 [0.00, 0.19]
	Ventral root-evoked EPSC rise-time (ms)	0.46 [0.36, 0.55]	0.07 [-0.07, 0.21]	9.1E-04 (0.86)	1.44E-02 (0.14)	0.04 [0.00, 0.24]
	Ventral root-evoked EPSC decay-time (ms)	4.58 [2.64, 6.53]	0.62 [-2.41, 3.66]	8.27 (0.92)	0.71 (0.08)	0.00 [0.00, 0.15]
Delayed firing motoneurons	Ventral root-evoked EPSC rise-time (ms)	0.50 [0.42, 0.58]	0.06 [-0.06, 0.18]	1.54E-02 (0.58)	1.13E-02 (0.42)	0.02 [0.00, 0.11]
	Ventral root-evoked EPSC decay-time (ms)	5.65 [4.69, 6.61]	-0.48 [-1.89, 0.94]	1.75 (0.47)	1.94 (0.53)	0.00 [0.00, 0.07]
Renshaw cells	BQA probability of release [Ca2+] = 1mM	0.19 [0.08, 0.30]	0.10 [-0.08, 0.28]	1.16E-02 (0.65)	8.97E-03 (0.35)	0.06 [0.00, 0.28]
	BQA probability of release [Ca2+] = 2mM	0.58 [0.40, 0.76]	0.26 [-0.03, 0.55]	3.14E-02 (0.43)	4.21E-03 (0.57)	0.15 [0.00, 0.39]

CI - confidence interval; ICC - intraclass correlation coefficient; EPSC - excitatory postsynaptic current; BQA - Bayesian Quantal Analysis

Table S25 – Linear mixed model fixed and random-effects variables and partial eta squared (η_p^2) for electrophysiological parameters obtained from ventral horn-ablated spinal cord slices

In vitro ventral horn-ablated spinal cord						
		Fixed effects Estimate [95% CI]		Random effects σ^2 (ICC)		Effect size
	Parameter	WT (intercept)	mSOD1	Animal	Residuals	η_p^2 [90% CI]
L4 segment L4 and L5 stimulation	Dorsal root-evoked IPSC paired-pulse ratio	1.05 [1.01, 1.10]	0.03 [-0.04, 0.09]	4.71E-03 (0.51)	4.44E-03 (0.49)	0.01 [0.00, 0.06]
	Dorsal root-evoked IPSC rise-time (ms)	0.55 [0.49, 0.60]	-0.05 [-0.13, 0.04]	8.91E-03 (0.69)	3.97E-03 (0.31)	0.01 [0.00, 0.07]
	Dorsal root-evoked IPSC decay-time (ms)	4.80 [4.14, 5.46]	-0.81 [-1.78, 0.16]	1.13 (0.58)	0.83 (0.42)	0.03 [0.00, 0.10]
L5 segment L4 and L5 stimulation	Dorsal root-evoked inhibition scaled (%)	179 [-60, 418]	164 [-174, 503]	21.7E04 (0.95)	12.2E03 (0.05)	0.01 [0.00, 0.04]
L4 segment L4 root stimulation	Post-activation depression (ratio)	0.83 [0.78, 0.87]	-0.02 [-0.10, 0.05]	5.35E-03 (0.58)	3.931E-03 (0.42)	0.00 [0.00, 0.02]
	Dorsal root-evoked IPSC paired-pulse ratio	1.05 [1.00, 1.10]	6.38E-03 [-0.06, 0.08]	5.54E-03 (0.66)	2.84E-03 (0.34)	0.00 [0.00, 0.04]
	Dorsal root-evoked IPSC rise-time (ms)	0.55 [0.49, 0.61]	-0.07 [-0.16, 0.03]	9.76E-03 (0.69)	4.36E-06 (0.31)	0.04 [0.00, 0.15]
L4 segment L5 root stimulation	Dorsal root-evoked excitation (nS)	35 [23, 47]	-6 [-24, 12]	297 (0.31)	650 (0.69)	0.01 [0.00, 0.06]
	Dorsal root-evoked EPSC rise-time (ms)	0.39 [0.35, 0.42]	0.02 [-0.03, 0.07]	3.08E-0.3 (0.47)	3.49E-0.3 (0.53)	0.01 [0.00, 0.06]
	Post-activation depression (ratio)	0.81 [0.75, 0.87]	0.05 [-0.12, 0.04]	5.10E-0.3 (0.41)	7.34E-0.3 (0.59)	0.02 [0.00, 0.11]
	Dorsal root-evoked IPSC paired-pulse ratio	1.05 [0.99, 1.10]	0.03 [-0.05, 0.11]	4.78E-0.3 (0.47)	5.39E-0.3 (0.53)	0.01 [0.00, 0.12]
	Dorsal root-evoked IPSC rise-time (ms)	0.53 [0.48, 0.57]	-0.02 [-0.10, 0.05]	3.82E-0.3 (0.48)	4.20E-0.3 (0.52)	0.01 [0.00, 0.11]
	Dorsal root-evoked IPSC decay-time (ms)	4.68 [4.02, 5.35]	-1.15 [-2.09, -0.20]	0.634 (0.43)	0.833 (0.57)	0.12 [0.01, 0.27]
L5 segment L4 root stimulation	Dorsal root-evoked excitation (nS)	30 [22, 38]	10 [-2, 22]	173 (0.32)	374 (0.68)	0.02 [0.00, 0.08]
	Dorsal root-evoked inhibition scaled (%)	191 [-129, 511]	169 [-267, 604]	281E03 (0.96)	11E03 (0.04)	0.01 [0.00, 0.06]
L5 segment L5 root stimulation	Dorsal root-evoked EPSC rise-time (ms)	0.40 [0.36, 0.43]	-0.02 [-0.07, 0.03]	3.43E-03 (0.39)	5.45E-03 (0.61)	0.00 [0.00, 0.04]

	Dorsal root-evoked IPSC rise-time (ms)	0.49 [0.45, 0.53]	0.02 [-0.04, 0.08]	4.50E-03 (0.54)	3.86E-03 (0.46)	0.00 [0.00, 0.05]
	Dorsal root-evoked IPSC decay-time (ms)	4.85 [4.27, 5.43]	-0.16 [-0.97, 0.66]	0.66 (0.34)	1.26 (0.66)	0.00 [0.00, 0.04]

CI - confidence interval; ICC - intraclass correlation coefficient; EPSC - excitatory postsynaptic current; IPSC - inhibitory postsynaptic current; BQA - Bayesian Quantal Analysis

Table S26 – Linear mixed model fixed and random-effects variables and partial eta squared for quantal size estimated from Bayesian Quantal Analysis (BQA) experiments obtained from *in vitro* ventral horn ablated spinal cords and conductance of recurrent inhibition estimated from *in vivo* motoneuron recordings

In vitro motoneuron recordings from ventral horn-ablated spinal cords					
	Fixed effects Estimate [95% CI]		Random effects σ^2 (ICC)		Effect size
Parameter	WT (intercept)	mSOD1	Animal	Residuals	η_p^2 [90% CI]
Quantal size scaled (%)	1.45 [0.89, 2.01]	0.07 [-0.80, 0.94]	0.49 (0.65)	2.19 (0.35)	0.00 [0.00, 0.03]
In vivo motoneuron recordings					
Recurrent inhibition (nS)	28 [3, 53]	10 [-25, 45]	803 (0.65)	440 (0.35)	0.00 [0.00, 0.12]
Recurrent inhibition scaled (%)	11 [4, 17]	-2 [-11, 7]	36 (0.31)	79 (0.69)	0.01 [0.00, 0.10]

CI - confidence interval; ICC - intraclass correlation coefficient;
